# An investigation of spatial-temporal patterns and predictions of the coronavirus 2019 pandemic in Colombia, 2020–2021

**DOI:** 10.1371/journal.pntd.0010228

**Published:** 2022-03-04

**Authors:** Amna Tariq, Tsira Chakhaia, Sushma Dahal, Alexander Ewing, Xinyi Hua, Sylvia K. Ofori, Olaseni Prince, Argita D. Salindri, Ayotomiwa Ezekiel Adeniyi, Juan M. Banda, Pavel Skums, Ruiyan Luo, Leidy Y. Lara-Díaz, Raimund Bürger, Isaac Chun-Hai Fung, Eunha Shim, Alexander Kirpich, Anuj Srivastava, Gerardo Chowell

**Affiliations:** 1 Department of Population Health Sciences, School of Public Health, Georgia State University, Atlanta, Georgia, United States of America; 2 Department of Biostatistics, Epidemiology and Environmental Health Sciences, Jiann-Ping Hsu College of Public Health, Georgia Southern University, Statesboro, Georgia, United States of America; 3 Department of Computer Science, College of Arts and Sciences, Georgia State University, Atlanta, Georgia, United States of America; 4 Centro de Investigación en Ingeniería Matemática (CI^2^MA) and Departamento de Ingeniería Matemática, Universidad de Concepción, Concepción, Chile; 5 Department of Mathematics and Integrative Institute of Basic Sciences, Soongsil University, Seoul, Republic of Korea; 6 Department of Statistics, Florida State University, Tallahassee, Florida, United States of America; University of Hong Kong, HONG KONG

## Abstract

Colombia announced the first case of severe acute respiratory syndrome coronavirus 2 on March 6, 2020. Since then, the country has reported a total of 5,002,387 cases and 127,258 deaths as of October 31, 2021. The aggressive transmission dynamics of SARS-CoV-2 motivate an investigation of COVID-19 at the national and regional levels in Colombia. We utilize the case incidence and mortality data to estimate the transmission potential and generate short-term forecasts of the COVID-19 pandemic to inform the public health policies using previously validated mathematical models. The analysis is augmented by the examination of geographic heterogeneity of COVID-19 at the departmental level along with the investigation of mobility and social media trends. Overall, the national and regional reproduction numbers show sustained disease transmission during the early phase of the pandemic, exhibiting sub-exponential growth dynamics. Whereas the most recent estimates of reproduction number indicate disease containment, with R_t_<1.0 as of October 31, 2021. On the forecasting front, the sub-epidemic model performs best at capturing the 30-day ahead COVID-19 trajectory compared to the Richards and generalized logistic growth model. Nevertheless, the spatial variability in the incidence rate patterns across different departments can be grouped into four distinct clusters. As the case incidence surged in July 2020, an increase in mobility patterns was also observed. On the contrary, a spike in the number of tweets indicating the stay-at-home orders was observed in November 2020 when the case incidence had already plateaued, indicating the pandemic fatigue in the country.

## Author summary

As the COVID-19 pandemic continues to spread across Colombia, studies highlighting the intensity of the pandemic have become imperative for appropriate resource allocation and informing public health policies. In this study we utilize mathematical models to infer the transmission dynamics of SARS-CoV-2 at the regional and national level as well as short-term forecasting of the COVID-19 epidemic trajectory. Moreover, we examine the geographic heterogeneity of the COVID-19 case incidence in Colombia along with the analysis of mobility and social media trends in relation to the observed COVID-19 case incidence in the country. The estimates of reproduction numbers at the national and regional level show a decline in disease transmission as of October 31, 2021. Moreover, the 30-day ahead short-term forecasts for the most recent time-period (October 2-October 31, 2021) generated using the mathematical models needs to be interpreted with caution as the single peak models point towards a sustained decline in case incidence contrary to the sub-epidemic wave model. Nevertheless, the spatial analysis in Colombia shows distinct variations in incidence rate patterns across different departments which can be grouped into four distinct clusters. Moreover, the social media and mobility trends also explain the occurrence of case resurgences over time.

## Introduction

Ever since the detection of the first cluster of severe acute respiratory syndrome coronavirus 2 (SARS-CoV-2) in December 2019, the coronavirus disease 2019 (COVID-19) pandemic continues to threaten the world [1]. As this pandemic gears to continue in the third year, it has already resulted in devastating global socioeconomic and political consequences [2]. The virus’s novelty and complex transmission dynamics have resulted in non-pharmaceutical public health measures including social distancing mandates and intermittent lockdowns as the primary weapons to fight COVID-19 [3]. Moreover, as new variants of SARS-CoV-2 continue to emerge amidst vaccination campaigns globally, it remains unclear how the COVID-19 pandemic will unfold [4,5]. Despite the uncertainty in the development of the pandemic, SARS-CoV-2 continues to exert substantial morbidity and mortality burden surpassing 246 million confirmed cases and nearly 5 million associated deaths worldwide as of October 31, 2021 [6]. The mortality and morbidity of the COVID-19 pandemic continues to overwhelm the health care systems of many nations including the United States, Colombia, Mexico, Brazil and Argentina in the Americas [7].

As the third COVID-19 wave strikes globally, it remains imperative to understand the dynamics of disease transmission and the potential role of mitigation strategies at different spatial scales including the municipality, regional or departmental level in order to contain the virus [8]. Assessing the geographical patterns of the cases and identifying the COVID-19 impact at the regional level can provide valuable insights regarding the exposure to the virus and the distribution of public health resources required to mitigate its spread [9,10]. Pandemic emergency response which includes the implementation and lifting of dynamic lockdowns [11], social distancing, mask mandates, population testing, and provision of vaccinations has varied across countries in the same region and in different cities within the same country [12]. As with Latin America, one of the epicenters of the pandemic [13], the evolution of the pandemic and the quality of public health responses have varied across nations. While Chile aimed to mitigate the virus to slow down the disease and reduce peak in health care demand, Brazil, Ecuador, and Colombia’s objective was to contain the spread of the virus by minimizing the risk of transmission from infected to non-infected individuals to stop the outbreak [14,15]. Despite aiming to contain virus transmission in the country, Colombia became the second country in Latin America and eighth globally to reach one million cases by the end of October 2020 [16]. Colombia has also seen the fastest increase in total COVID-19 associated deaths compared to other Latin American countries as reported by the end of the year 2020 [14].

In Latin America, where the first confirmed COVID-19 case was reported in Brazil on February 26, 2020 [17], the intensity and diversity of social distancing efforts have varied widely. Since the detection of the first imported confirmed case on March 6, 2020, Colombia has observed three epidemic peaks [13,18]. As the virus disseminated throughout the country, initial prevention recommendations provided between March 9–11, 2020 from the local government authorities conflicted with those at the national level [19], creating confusion amongst the public about the preventive measures required to protect themselves [19]. Therefore, the case incidence continued to rise leading to the declaration of the national emergency on March 12, 2020 [19]. As a part of the prompt escalation of measures to prevent a full-blown outbreak most educational institutions were closed by March 16, 2020, followed by the closure of the country borders the next day [19].

As cases continued to increase rapidly, domestic and international flights were suspended on March 23, 2020 [19]. Subsequently, to limit virus transmission within the country, a nationwide mandatory quarantine was implemented on March 24, 2020. Meanwhile a phased reopening of the economy was initiated as early as April 27, 2020, under strict protocols, to support the country’s dwindling economy [19].

The evolution of the pandemic in Colombia has justified the five extensions of the mandatory quarantine that lasted until August 31, 2020 [20]. At this point, the country transitioned into a period of “selective isolation with responsible individual distancing” as the daily incidence in the country’s main cities including Bogotá, Medellín, Cali, Bucaramanga and Pasto levelled off and eventually leaned towards a downward trend [21,22]. Moreover, Barranquilla, Cartagena, Leticia and Quibdó had overcome the worst part of the first wave by August 25, 2020 [21]. The chosen selective isolation strategy prioritized tracing of suspected cases, those with infection, and their contacts, while reactivating the social and economic life of the country. As the cases continued to increase in the subsequent months, the government imposed and lifted dynamic lockdowns in multiple cities across the country. By the end of year 2020, COVID-19 cases were mainly concentrated in Bogotá (314,745), the capital city of Colombia followed by Antioquia (163,952), and Valle del Cauca (81,101) [23].

As the pandemic entered its second year, a second wave of COVID-19 was detected, primarily fueled by the P.1 (Gamma) variant of SARS-CoV-2 initially circulating in Brazil [24]. Soon after, the government announced a mass vaccination strategy that began on February 20, 2021 [25]. Around the same time, on February 25, 2021, the president of Colombia extended the duration of the national health emergency to last until May 31, 2021 [26]. Amidst delayed vaccine acquisition and slow vaccine distribution in the country, the cases continued to rise in some cities like Bogota and Barranquilla [27,28]. This resulted in the implementation of curfews in the cities and municipalities followed by the opening of businesses based on the occupancy rates of the intensive care units (ICU) in the hospitals beginning March 25, 2021 [27]. From March to June 2021, Colombia experienced a massive third wave of the COVID-19 pandemic, which evolved into a two stage peak-within-a-peak surge [28]. The month of May 2021 was reported to be the deadliest month resulting in an average of ~20,000 cases and 500 deaths per day [29]. While the impact of COVID-19 pandemic was not uniform across the entire country, Bogotá, Antioquia and Valle del Cauca have been the hardest hit areas in Colombia [30].

The Colombian government’s response to contain the COVID-19 pandemic has been declared the third worst out of 116 countries evaluated by the Lowy Institute in Australia, only ahead of Mexico and Peru [31]. Despite being one of the first countries in Latin America to offer diagnostic tests for COVID-19 [32], the testing and vaccination rate for Colombians remains low [33], with 0.79 tests per 1000 people per day and 0.39 vaccine doses administered per 100 people as of October 31, 2021. [34]. Hence, the country stands with a total of 5,002,387 cases and 127,258 deaths as of October 31, 2021 [30]. The factors contributing to the current COVID-19 outbreak are myriad. However, three distinguishable events have particularly interacted synergistically to add to the complexity of the pandemic in Colombia. These include the COVID-19 outbreaks in prisons and nursing homes that affected the vulnerable communities of the society [35,36] and the April 2021 Colombian protests provoked by the government policy proposals [28]. The Colombian prisons in Cali, Villavicencio, and Bogotá were severely impacted by the COVID-19 pandemic due to over-crowding, inadequate medical supplies and unhygienic conditions of facilities, which led to many infected inmates [36].

The COVID-19 pandemic in Colombia presents complex risk dynamics of SARS-CoV-2 transmission with a simultaneous interplay of epidemiological, behavioral, and political factors. Hence assessing the temporal variation of transmission in near real time becomes vital to evaluate the effectiveness of intervention strategies and possible reopening of the economy. Moreover, forecasting the COVID-19 trajectory can help understand the trends of the disease and estimate its potential burden. As the epidemic trajectory of the COVID-19 pandemic continues to unfold we forecast the COVID-19 trajectory in near-real time utilizing the mathematical models that have been validated for previous infectious disease outbreaks such as Ebola, Zika and the COVID-19 pandemic [37–40]. Moreover, we specifically investigate the transmission dynamics of SARS-CoV-2 at the national and regional levels. We augment the analysis by investigating the spatial epidemic curves that allow us to analyze geographic heterogeneity that is lost while evaluating the aggregated epidemic curves and examine the social media and mobility trends concerning the implementation of lockdowns.

## Methods

### Data

Six sources of data are analyzed in this study. A brief description of the data sets is provided as follows:

(i) Case incidence data

Case incidence data based on the dates of symptom onset is used to generate the epidemic curve, scaled incidence curve (see S1 Text), short-term forecasts of the COVID-19 pandemic, assess the spatial heterogeneity in Colombia and estimate the national and regional reproduction numbers. Specifically, we utilize publicly available time series of laboratory-confirmed cases based on the dates of symptom onset for the national and regional COVID-19 case incidence. These data are retrieved from the Colombian Ministry of Health as of October 31, 2021 [23]. Colombian departments and their four main districts; Barranquilla, Bogotá, Cartagena, and Santa Marta can be grouped into five regions as follows: the Amazon, the Andean, the Orinoquía, the Caribbean and the Pacific [41]. To evaluate the spatial heterogeneity and estimate the regional reproduction numbers, for simplicity we assume a regional level distribution as follows (S1 Fig):

Amazon region: Amazonas, Caquetá, Guainía, Guaviare, Putumayo and Vaupés.
Andean region: Antioquia, Boyacá, Bogotá, Caldas, Cauca, Cundinamarca, Huila, Norte De Santander, Quindío, Risaralda, Santander, and Tolima.
Orinoquía region: Arauca, Casanare, Meta, and Vichada
Caribbean region: Atlántico, Bolívar, Cesar, Córdoba, La Guajira, Magdalena, San Andrés, Sucre, Barranquilla, Cartagena, and Santa Marta
Pacific region: Chocó, Nariño, and Valle Del Cauca

Based on the geographical distribution of the departments, Nariño, Valle Del Cauca, Guainía, and Cauca appear in two regions each. However, we include them in one region as stated above, to avoid the duplication of data for our analysis.

(ii) Mortality data

COVID-19 mortality data based on the date of death is retrieved from the Colombian Ministry of Health as of October 31, 2021 [23]. Mortality data is used to generate the national short-term forecast of the COVID-19 pandemic and estimate the national reproduction number.

(iii) Genomic data

To estimate the reproduction number from the genomic data, from the 1855 samples available as of June 30, 2021, 136 SARS-CoV-2 genome samples for Colombia are obtained from the “Global Initiative on Sharing Avian Influenza Data” (GISAID) repository [42] between February 27 and April 5, 2020 to estimate the reproduction number.

(iv) Mobility trends data

Two sources of mobility data are analyzed at the national level from March 6, 2020, to October 31, 2021. These data were retrieved as of October 31, 2021.

Apple’s mobility data is a set of data that summarizes the number of requests made to Apple Maps for directions. The data are categorized by countries/regions, sub-regions, and cities. Apple has released the data for the three modes of human mobility: driving, walking and public transit. These data are published publicly by Apple’s mobility trends reports [43]. These aggregated and anonymized data are updated daily and includes the relative volume of directions requests per country compared to the baseline volume on January 13, 2020. To emphasize on the change rather than the gross number of requests, Apple has normalized all the data. The number of direction requests for each country or city and the type of direction request (driving, walking and public transit) has been set to 100 for January 13, 2020. All subsequent data are relative to the starting point.

Google’s mobility data [44] is retrieved from users who have turned on the location history in their Google account. Google’s mobility data shows the changes in mobility in each geographic and administrative region for visits to different places, such as grocery stores, parks, and recreation spots. The baseline day represents the normal value on the day of the week. The baseline day is the median value from the 5-week period from January 3, 2020, to February 6, 2020.

(v) Twitter data

We retrieved data from the publicly available Twitter data set of the COVID-19 chatter from March 12, 2020 to October 31, 2021 [45]. A detailed description of Twitter data acquisition and processing is provided in a prior study [45].

### Modeling framework for forecast generation

We utilize and compare three dynamic phenomenological growth models to generate short-term (i.e., 30-day ahead) forecasts for Colombia. These models have been applied to various infectious diseases including SARS, foot and mouth disease, Ebola [46–48] and the current COVID-19 outbreak [49–51]. The phenomenological growth models applied in this study include the two models defined by one scalar differential equation namely the generalized logistic growth model (GLM) [47] and the Richards growth model [52]. Additionally we apply the sub-epidemic wave model [46] which captures diverse epidemic trajectories such as the multimodal outbreaks. The forecasts obtained from these dynamic growth models can assess the potential scope of the pandemic in near real-time, provide insights on the contribution of disease transmission pathways, predict the impact of control interventions, and evaluate optimal resource allocation to inform public health policies. A detailed description of these models is provided in S2 Text.

### Model calibration and forecasting approach

We utilize the national and regional level case incidence data retrieved from the Colombian Ministry of Health based on the dates of symptom onset as of October 31, 2021. We also utilize the national level mortality data based on the date of death as of October 31, 2021. Each forecast is fitted to the daily case counts based on the dates of symptom onset and daily death counts based on the date of death between July 4, 2021, and October 1, 2021 (90 days calibration period) to conduct a 30-day ahead short-term forecast for each model. The data from October 2, 2020, to October 31, 2021, is utilized to assess the performance of our 30-day ahead short-term forecasts. Details of the model calibration and forecasting approach are provided in S3 Text.

### Performance metrics

The following five performance metrics are used to assess the quality of our model fit and the 30-day ahead short-term forecasts: the mean absolute error (MAE) [53]; the root mean squared error (RMSE) [54]; the coverage of the 95% prediction intervals (95% PI) [54]; the mean interval score (MIS) [54] and the weighted interval score (WIS) [55]; for each of the three models: GLM; Richards model; and the sub-epidemic wave model. A detailed description of the performance metrics is provided in S4 Text.

### Reproduction number

In order to understand the transmission dynamics of COVID-19, we estimate the effective reproduction number, *R*_*t*_, for the early ascending phase from February 27, 2020, to March 27, 2020, and the effective reproduction number *R*_*t*_ throughout the pandemic for the national and regional COVID-19 epidemic curves. The effective reproduction number, *R*_*t*_, is the key parameter that characterizes the average number of secondary cases generated by a primary case at calendar time (*t*) during the outbreak. This quantity is crucial for identifying the magnitude of public health interventions required to contain an epidemic [56–58]. The estimates of *R*_*t*_ indicate whether widespread disease transmission continues (*R*_*t*_>1) or disease transmission declines (*R*_*t*_<1). Therefore, to contain an outbreak, it is vital to maintain *R*_*t*_<1. We utilize the case incidence, mortality, and the genomic data to estimate the reproduction numbers using three rigorous methods to compare the estimates of *R*_*t*_ obtained from each method and data source.

### Effective reproduction number *R*_*t*_, using the generalized growth model (GGM)

We estimate the national and regional reproduction numbers by calibrating GGM to the early growth phase of the pandemic [59], from February 27, 2020 to March 27, 2020. The description of the GGM is provided in S2 Text. The generation interval of SARS-CoV-2 is modeled with the assumption of a gamma distribution with a mean of 5.2 days and a standard deviation of 1.72 days [60]. We first characterize the daily incidence of local cases using the GGM. The progression of local incidence cases by dates of symptom onset, *I*_*i*_, is simulated using the calibrated GGM model and account for the daily series of imported cases by dates of symptom onset, *J*_*i*_, into the renewal equation to estimate the effective reproduction, $R_{t_{i}}$ as

$$R_{t_{i}}=\frac{I_{i}}{\sum_{j=0}^{i}{(I}_{i-j}+\alpha J_{i-j})\rho_{j}}.$$

The factor *J*_*i*_ represents the imported cases at time *t*_*i*_, *I*_*i*_ denotes the local case incidence at calendar time *t*_*i*_ and *ρ*_*j*_ represents the discretized probability distribution of the generation interval. The factor 0≤*α*≤1 represents the relative contribution of imported cases to secondary disease transmission. We perform sensitivity analysis by setting *α* = 0.15, *α* = 1.00 and *α* = 0.00 to assess the relative contribution of the imported cases to the secondary disease transmission [40]. The numerator represents the total new cases *I*_*i*_, and the denominator represents the total number of primary cases that contributes to the generation the new cases *I*_*i*_ (i.e., as secondary cases) at time *t*_*i*_. Hence, *R*_*t*_, represents the average number of secondary cases generated by a single case at calendar time *t*. The uncertainty bounds around the curve of $R_{t_{i}}$ are derived directly from the 300 bootstrap samples generated from the GGM model with estimated parameters ($\hat{r_{i}},\;\hat{p_{i}}$) where i = 1,2, …S. We assume a negative binomial error structure where the variance is assumed to be three times the mean based on the noise of the data [61]. This method is utilized to derive the early estimates of reproduction number and has been employed in several prior studies [50,61,62].

Since the national and regional epidemic curves have a distinct date of onset according to the seeding of the first local case, therefore, *R*_*t*_ for the national and regional level is estimated based on the date of onset of the first local case. For the national and regional epidemic curves, we estimate *R*_*t*_ for the first 30 days (Table 1).

**Table 1:** Dates for *R*_*t*_ estimation for the national and regional epidemic curves for the first 30 epidemic days.

Region	Dates (2020) Month/DD
National	February 28- March 28
Orinoquía region	March 17-April 17
Amazon region	March 25-April 23
Caribbean region	February 29-March 29
Andean region	March 01-March 30
Pacific region	February 28-March 28

### Effective reproduction number *R*_*t*_, using the Cori et al. method

We estimate the regional and national effective reproduction numbers (also known as the time dependent instantaneous reproduction numbers) using the local and imported case incidence data based on the date of symptoms onset as of October 31, 2021. The total number of incident cases arising at time step t, *I*_*t*_, is the sum of the number of local incident cases ($I_{t}^{local}$) and imported incident cases ($I_{t}^{imported}$). Hence,

$$I_{t}=I_{t}^{local}+I_{t}^{imported}.$$

We assume that if the imported cases exist, they can be distinguished from the local cases through epidemiological investigations so that $I_{t}^{local}$ and $I_{t}^{imported}$ can be observed at each time step [63,64].

The time dependent (instantaneous) *R*_*t*_ is defined as the ratio of the number of new locally infected cases generated at calendar time *t* ($I_{t}^{local}$), and the total infectiousness across all infected individuals at time *t* given by $\sum_{s=1}^{t}{(I}_{t-s}^{local}+I_{t-s}^{imported})w_{s}=\sum_{s=1}^{t}I_{t-s}w_{s}$ [65,66]. Hence *R*_*t*_ can be written as

$$R_{t}=\frac{I_{t}^{local}}{\sum_{s=1}^{t}I_{t-s}w_{s}}.$$

In this equation, the factor *w*_*s*_ represents the infectivity function, which is the infectivity profile of the infected individual. The infectivity function is dependent on the time since infection (*s)*, but is independent of the calendar time (*t*) [67,68].

The term $\sum_{s=1}^{t}I_{t-s}w_{s}$ describes the sum of infection incidence up to time step *t* − 1, weighted by the values of infectivity function *w*_*s*_. The distribution of the generation time can be applied to approximate *w*_*s*_, however, since the time of infection is a rarely observed event, measuring the distribution of generation time becomes difficult [65]. Therefore, the time of symptom onset is usually used to estimate the distribution of the serial interval (SI), which is defined as the time interval between the dates of symptom onset among two successive cases in a disease transmission chain [69].

The infectiousness of a case is a function of the time since infection, which is proportional to *w*_*s*_ if the timing of infection in the primary case is set as time zero of *w*_*s*_ and we assume that the generation interval equals the serial interval (SI). The SI is assumed to follow a gamma distribution with a mean of 5.2 days and a standard deviation of 1.72 days [60]. We also estimate *R*_*t*_ for the mortality curve by the date of death as of October 31, 2021. The analytical estimates of *R*_*t*_ are obtained within a Bayesian framework using the Cori et al. method, employing the EpiEstim package in R version 4.0.3. [69]. The values for *R*_*t*_ are estimated using 1-weekly sliding window. We report the median and 95% credible interval (CrI).

### Reproduction number, *R*, from the genomic analysis

To estimate the reproduction number for SARS-CoV-2 between February 27, 2020 and April 5, 2020 from the genomic data, 136 SARS-CoV-2 genome samples from Colombia and their sampling times are obtained from the GISAID repository [42,70]. Short sequences and sequences with significant number of gaps and non-identified nucleotides are removed. For clustering, they are complemented by the sequences from other geographical regions across the globe, downsampled to n = 1470 representative sequences using a country specific sub-sampling algorithm of Nextstrain. Briefly, the algorithm first chooses the sequences from the focal country, and then complements them with the sequences obtained from other geographical regions, prioritizing sequences from the geographical neighbors and the sequences that are genetically close to the focal sequences. This decreases the size of the genomic data set to allow for feasible phylogenetic inference. We use the sequence subsample from Nextstrain (www.nextstrain.org) global analysis as of July 4, 2021. These sequences are aligned to the reference genome obtained from the literature [71] using Muscle [72] and trimmed using Molecular Evolutionary Genetics Analysis version: 7 (Mega 7) [73].

The largest Colombian cluster that possibly corresponds to within-country transmissions has been identified using hierarchical clustering of sequences. Phylodynamics analysis of that cluster is carried out using Bayesian Evolutionary Analysis by Sampling Trees (BEAST) [74] version 2. We use a strict molecular clock and a tree prior with exponential growth coalescent. Markov Chain Monte Carlo (MCMC) sampling is run for 10,000,000 iterations, and the parameters are sampled every 1000 iterations. The results are acceptable if the effective sample sizes are above 200 for all parameters. The exponential growth rate, *f* estimated by BEAST is used to calculate the basic reproduction number, *R*_0_. We utilize the standard assumption that SARS-CoV-2 generation intervals (times between infection and onward transmission) are gamma-distributed [75]. In this case, *R*_0_ can be estimated as

$$R_{0}={\left(1+\frac{f\sigma^{2}}{\mu }\right)}^{\frac{\mu^{2}}{\sigma^{2}}},$$

where *μ* and *σ* are the mean and standard deviation of the gamma distribution, respectively. The values of *μ* = 5.2 days and *σ* = 1.72 days are taken from a prior study [60]. The formula for *R*_0_ is a strictly monotone transformation of $\hat{f}$, therefore it also transforms the 95% highest posterior density (HPD) intervals for *f* into those for *R*_0_.

### Spatial analysis

We quantify and statistically analyze the shapes of the incidence rate curves. Shape analysis of the national and regional incidence rate curves is performed following the analytical methodology as described in previous literature [76] including steps to pre-process the daily cumulative COVID-19 case data based on the dates of symptom onset. Then, we analyze the shapes of these rate curves to compare, cluster and summarize the incidence rates. The pre-processing steps applied to the COVID-19 daily count data are as follows:

- Smoothing: Cumulative case curves are smoothed over 10 days using the smooth function in the MATLAB. The smooth function returns a moving average of the elements of a vector using a fixed window length which is determined heuristically, and in the case of spatial analysis, it is 10 days. The window slides down the length of the vector, computing an average over the elements within each window.
- Time differencing: If *f*_*i*_(*t*) denotes the given cumulative number of confirmed cases for department *i* on day *t*, then the growth rate per day at time *t* is given by *g*_*i*_(*t*) = *f*_*i*_(*t*)−*f*_*i*_(*t*−1).
- Rescaling: Each curve is rescaled by dividing each *g*_*i*_ by the total number of confirmed cases.
- Smoothing: We then smooth the normalized curves again over a 5-day span using the smooth function in the MATLAB.

To identify the clusters by comparing the curves, we used a simple metric. For any two rate curves, *h*_*i*_ and *h*_*j*_, we compute the norm *||h*_*i*_ *−h*_*j*_*||*, where the double bars denote the *L*^*2*^ norm of the difference function, that is,

$$||hi-hj||=\sqrt{\sum_{t}{\left(h_{i}(t)-h_{j}(t)\right)}^{2}}.$$

To perform clustering of thirty six curves into smaller groups, we apply the dendrogram function in the MATLAB using the “ward” linkage as explained in reference [76]. The number of clusters is determined empirically based on the display of overall clustering results. After clustering the departments and administrative divisions into different groups, we derive the average curve for each cluster after using a time wrapping algorithm as performed in previous studies [76,77]. All analysis is conducted in MATLAB R2020b.

### Twitter data analysis

To observe any relationship between the COVID-19 cases based on the dates of symptom onset and the frequency of tweets indicating stay-at-home orders we used a public dataset of 9,233,913,414 unique tweets of the COVID-19 chatter [45]. The frequency of tweets indicating stay-at-home order is used to infer people’s compliance with the stay-at-home-orders implemented to avoid spread of the virus by practicing social distancing. Tweets indicate the number of the people in favor of lockdowns and show how these numbers have varied throughout the pandemic. We removed all retweets and tweets not in the Spanish language to obtain the plotted data. We also filtered the tweets using the following hashtags: #quedateencasa (stay-at-home), and #trabajardesdecasa (work-from-home), which are two of the most used hashtags when users refer to the COVID-19 pandemic and their engagement with health measures. Lastly, our analysis was restricted to the tweets that originated from Colombia. A set of 493,276 unique tweets were gathered between March 12, 2020, and October 31, 2021. The usage of Twitter in Colombia is less compared to other Latin American countries like Mexico and Chile [78], hence we observe a lower proportion of filtered tweets. We overlay the curve of filtered tweets and the epidemic curve in Colombia to observe any relation between the shape of the epidemic trajectory and the shape of curve for the frequency of filtered tweets during the established time-period. The correlation coefficient between the daily cases based on the dates of symptom onset and frequency of filtered tweets is also estimated using the "corrcoef” function in MATLAB version R2020b. This coefficient explains the linear dependency of the daily cases based on the dates of symptom onset and frequency of filtered tweets.

### Mobility data analysis

We utilize the R code developed by Healy [79] to analyze the time series data for Colombia from March 6, 2020 to October 31, 2021, for two modes of mobility: driving and walking derived from Apple’s mobility reports. We analyze the mobility trends to discover any commonality in pattern of the mobility curves with the epidemic curve of COVID-19. The time series for mobility requests is decomposed into trends, weekly and remainder components. The trend is obtained by applying locally weighted regression fitted to the data and the remainder is any residual leftover on any given day after accounting for the underlying trend and normal daily fluctuations. The analysis is performed using ‘covmobility’ package in R software, version 4.0.3.

We analyze the Google mobility trends from March 6, 2020, to October 31, 2021 for visits to the grocery stores and pharmacy, parks, transit stations, workplaces, and residential areas compared to the baseline which is the median value, for the corresponding day of the week, during the five weeks from January 3, 2020 to February 6, 2020. For each region category, the baseline is not a single value; it comprises of seven individual values. The same number of visitors on two different days of the week results in different percentage changes. The analysis is performed using ‘tidycovid19’ package and R software, version 4.0.3.

## Results

**Fig 1:**
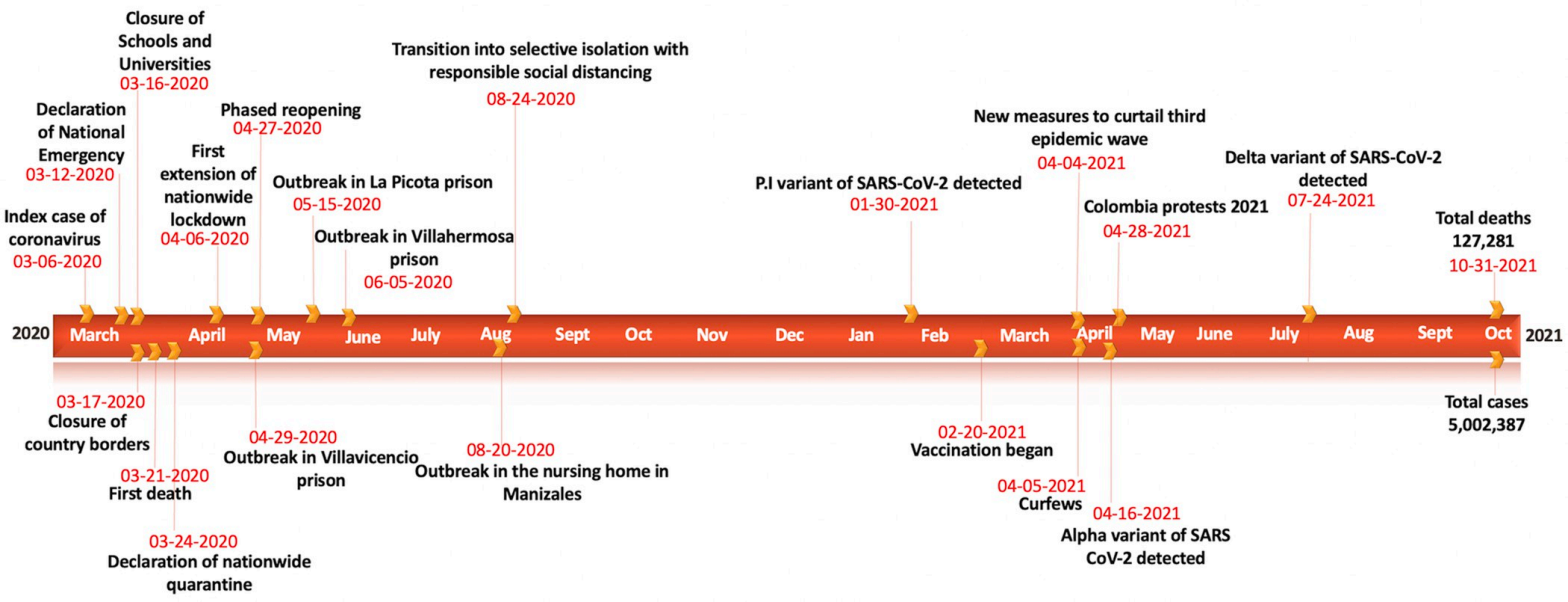
Timeline of the COVID-19 pandemic in Colombia as of October 31, 2021.

**Fig 2:**
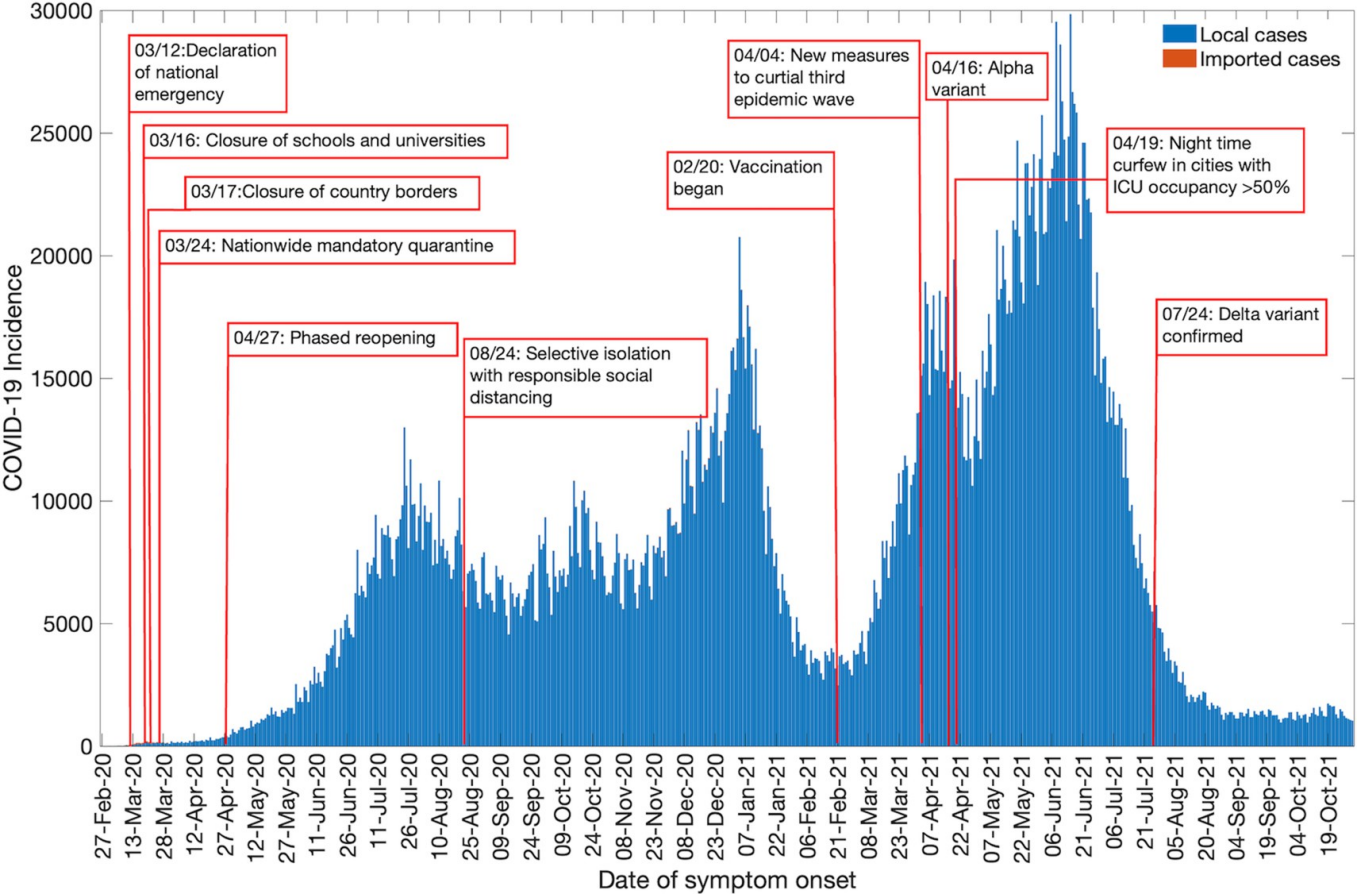
Epidemic curve of the COVID-19 pandemic in Colombia as of October 31, 2021.

A timeline showing the major events during the COVID-19 pandemic in Colombia is presented in Fig 1. As of October 31, 2021, Colombia has reported 4,537,370 cases based on the dates of symptom onset. The highest number of cases have been reported in the Andean region (2,935,282 cases) followed by the Caribbean region (888,914 cases), the Pacific region (518,203 cases), the Orinoquía region (140,549 cases) and the Amazon region (54,431 cases). The COVID-19 epidemic curve in Colombia shows a five-modal pattern with the first peak occurring in mid-July 2020 after the phased reopening of the country. It was followed by a small surge that occurred in mid-October 2020 after the implementation of selective isolation and responsible social distancing interventions. The second peak occurred at the beginning of January 2021 and the third protracted surge of the COVID-19 cases can be observed from March through June 2021 (Fig 2). The mortality curve (S2 Fig) for COVID-19 in Colombia also shows a three-modal pattern. The peaks for the mortality curve coincide with the three epidemic waves observed for the case incidence data. The first peak occurred in July 2020 followed by a second peak in January 2021 and a third larger peak from April through June 2021.

### Model calibration and forecasting performance

We compare the results from model calibration and 30-day ahead forecasting across three models: GLM, Richards and the sub-epidemic wave model. Model calibration, using 90 days of the epidemic data, between July 4, 2021 and October 1, 2021 shows that at the regional and national levels, the sub-epidemic model outperformed the GLM and Richards growth model in terms of the all five performance metrics (RMSE, MAE, MIS, WIS and 95% PI coverage) (Table 2) using the case incidence data. Therefore, the sub-epidemic wave model can be declared the most accurate model for the calibration period. The model fits exhibited sub-exponential growth dynamics [48,59] for three models (*p*~0.6–0.8) at the national and regional levels. The calibration performances for each region and the national data are listed in Table 2. Calibrating the three models to the mortality data also shows that the sub-epidemic model outperforms the other two models (S1 Table) and exhibits sub-exponential growth dynamics.

**Table 2:** Comparison of model performance metrics by calibrating the GLM, Richards and the sub-epidemic model for 90 epidemic days (July 4, 2021 to October 1, 2021) at the national and regional level. Higher 95% PI coverage and lower RMSE, MAE, WIS and MIS represent better performance. Best performing model is given in bold with the superscript “a”.

Regions	RMSE	MAE	MIS	95% PI	WIS
	GLM				
National	884.72	1315.3	18776	47.78	993.90
Pacific	97.36	152.17	1970.40	56.67	116.65
Caribbean	166.11	265.68	4457.80	61.10	207.89
Andean	467.69	738.12	**7787.10** ^**a**^	61.11	542.25
Amazon	**1.94** ^**a**^	11.70	86.04	**98.89** ^**a**^	8.15
Orinoquía	**19.13** ^**a**^	34.25	338.41	57.78	25.49
	Richards model				
National	1236.7	1643.5	31087	31.11	1323.2
Pacific	166.66	221.63	4186.9	31.11	180.51
Caribbean	300.60	372.66	9517.4	25.56	320.68
Andean	714.72	989.42	16221	37.78	771.77
Amazon	12.75	21.50	294.10	44.44	16.02
Orinoquía	40.90	53.87	1231.6	16.67	45.58
	Sub-epidemic wave model				
National	**442.6** ^**a**^	**298.15** ^**a**^	**1941.1** ^**a**^	**98.88** ^**a**^	**187.03** ^**a**^
Pacific	**93.18** ^**a**^	**62.48** ^**a**^	**314.25** ^**a**^	**96.67** ^**a**^	**93.18** ^**a**^
Caribbean	**134.47** ^**a**^	**99.02** ^**a**^	**666.6** ^**a**^	**91.11** ^**a**^	**64.98** ^**a**^
Andean	**353.9** ^**a**^	**219.76** ^**a**^	12710	**98.88** ^**a**^	**143.88** ^**a**^
Amazon	18.46	**10.61** ^**a**^	**85.39** ^**a**^	95.55	**6.87** ^**a**^
Orinoquía	36.42	**19.300** ^**a**^	**145.06** ^**a**^	**94.44** ^**a**^	**12.86** ^**a**^

In terms of the forecasting performance, again the sub-epidemic wave model performed better than the GLM and Richards model for the national and regional case incidence data (Table 3). The sub-epidemic model also outperformed the GLM and Richards model in the forecasting phase of the mortality curve (S2 Table). Hence, the sub-epidemic model can be considered the most accurate model to forecast the epidemic trajectory.

**Table 3:** Comparison of 30-day ahead forecasting performance (October 2, 2021 to October 31, 2021) by calibrating the GLM, Richards and the sub-epidemic model for 90 epidemic days (July 4, 2021 to October 1, 2021) at the national and regional level. Higher 95% PI coverage and lower RMSE, MAE, WIS and MIS represent better performance. Best performing model is given in bold with the superscript "a”.

Regions	RMSE	MAE	MIS	95% PI	WIS
	GLM				
National	1328.1	1312.5	**101300** ^**a**^	0	1037.8
Pacific	144.09	141.42	10682	0	115
Caribbean	499.71	487.00	30368	61.1	279.17
Andean	655.34	646.37	43420	0	609
Amazon	13.62	12.39	337.39	17.24	**4.24** ^**a**^
Orinoquía	20.35	18.97	1537.6	0	17
	Richards model				
National	1322.8	1307.2	130740	0	1038
Pacific	143.84	141.25	16478	0	115
Caribbean	500.28	487.14	45484	0	219
Andean	655.14	646.79	63713	0	609
Amazon	14.16	13.03	1075.2	0	6
Orinoquía	20.29	18.99	3214.3	0	17
	Sub-epidemic wave model				
National	**466.32** ^**a**^	**405.5** ^**a**^	26356000	**73.33** ^**a**^	**273.19** ^**a**^
Pacific	**41.746** ^**a**^	**36.03** ^**a**^	**210.01** ^**a**^	**100** ^**a**^	**20.44** ^**a**^
Caribbean	**135.15** ^**a**^	**105.5** ^**a**^	**676.2** ^**a**^	**86.67** ^**a**^	**69.68** ^**a**^
Andean	**109.38** ^**a**^	**90.69** ^**a**^	**733.3** ^**a**^	**100** ^**a**^	**142.9** ^**a**^
Amazon	**11.68** ^**a**^	**10.61** ^**a**^	**60.3** ^**a**^	**66.67** ^**a**^	8
Orinoquía	**7.266** ^**a**^	**5.80** ^**a**^	**45.6** ^**a**^	**100** ^**a**^	**3.90** ^**a**^

### 30-day ahead forecasts

**Fig 3:**
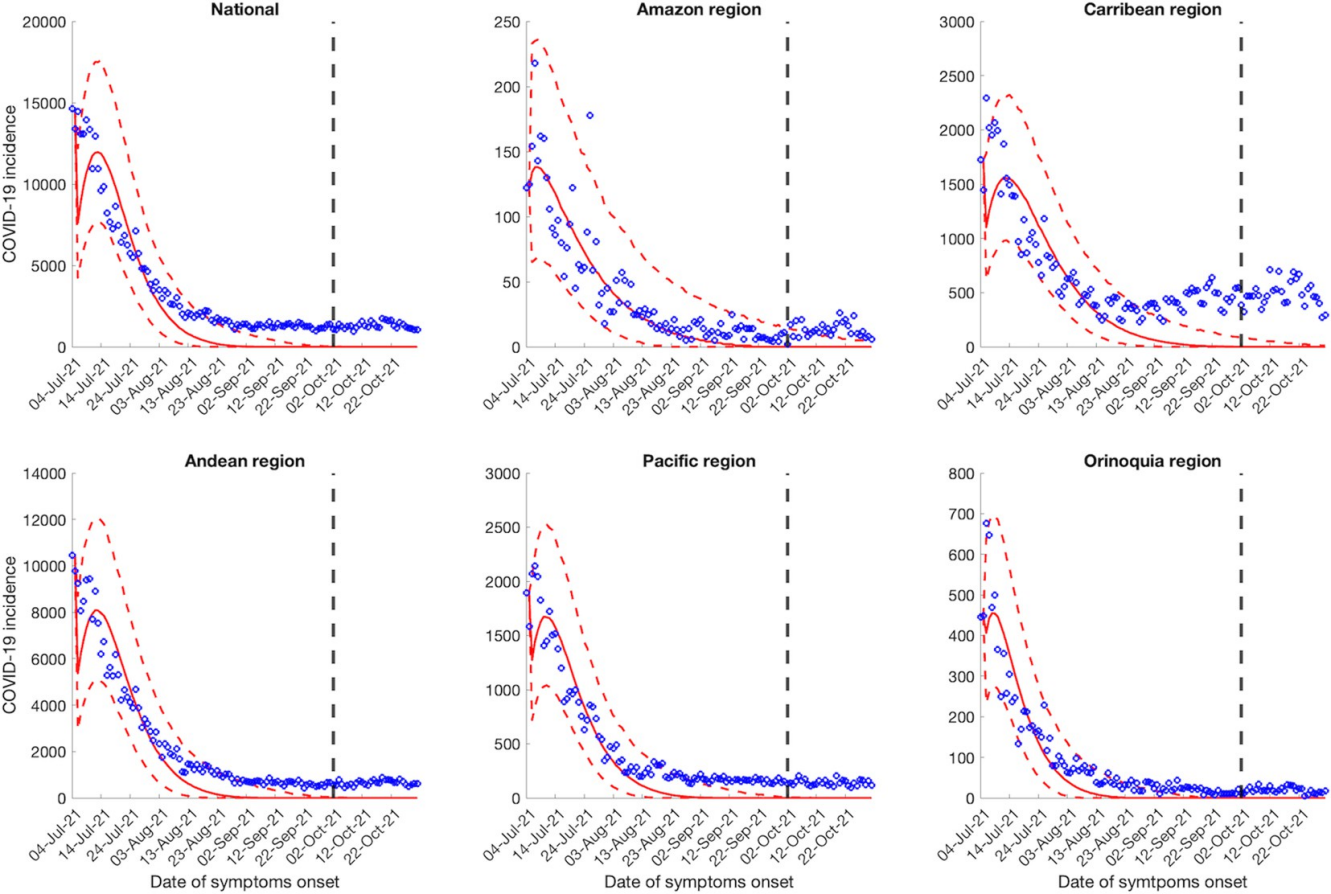
30-days ahead forecasts of the national and regional COVID-19 epidemic curves in Colombia by calibrating the GLM model from July 4, 2021 to October 1, 2021. The blue circles correspond to the data points; the solid red line indicates the best model fit, and the red dashed lines represent the 95% prediction interval. The vertical black dashed line represents the time of the start of the forecast period.

**Fig 4:**
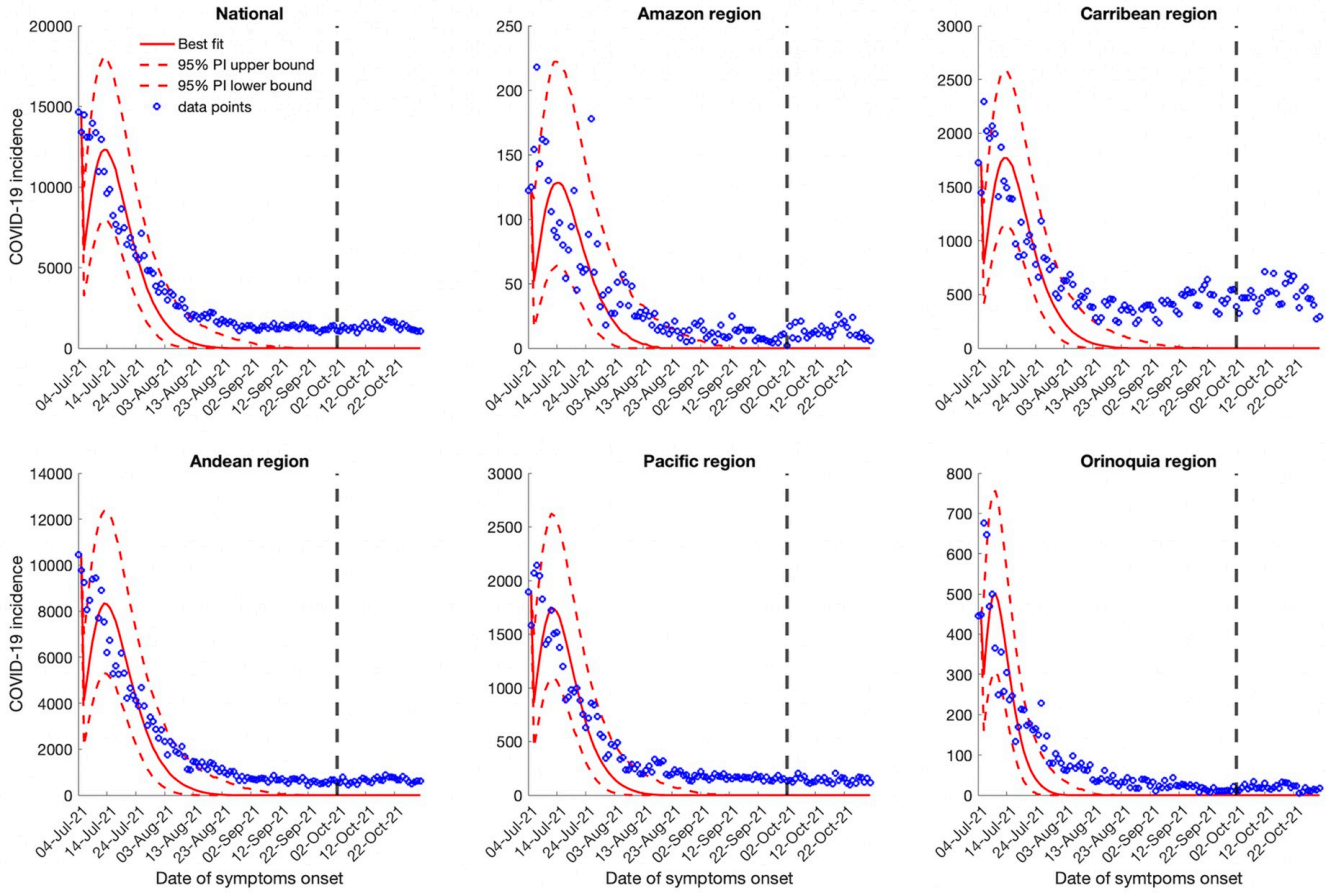
30-days ahead forecast of the national and regional COVID-19 epidemic curves in Colombia by calibrating the Richards model from July 4, 2021 to October 1, 2021. The blue circles correspond to the data points; the solid red line indicates the best model fit, and the red dashed lines represent the 95% prediction interval. The vertical black dashed line represents the time of the start of the forecast period.

**Fig 5:**
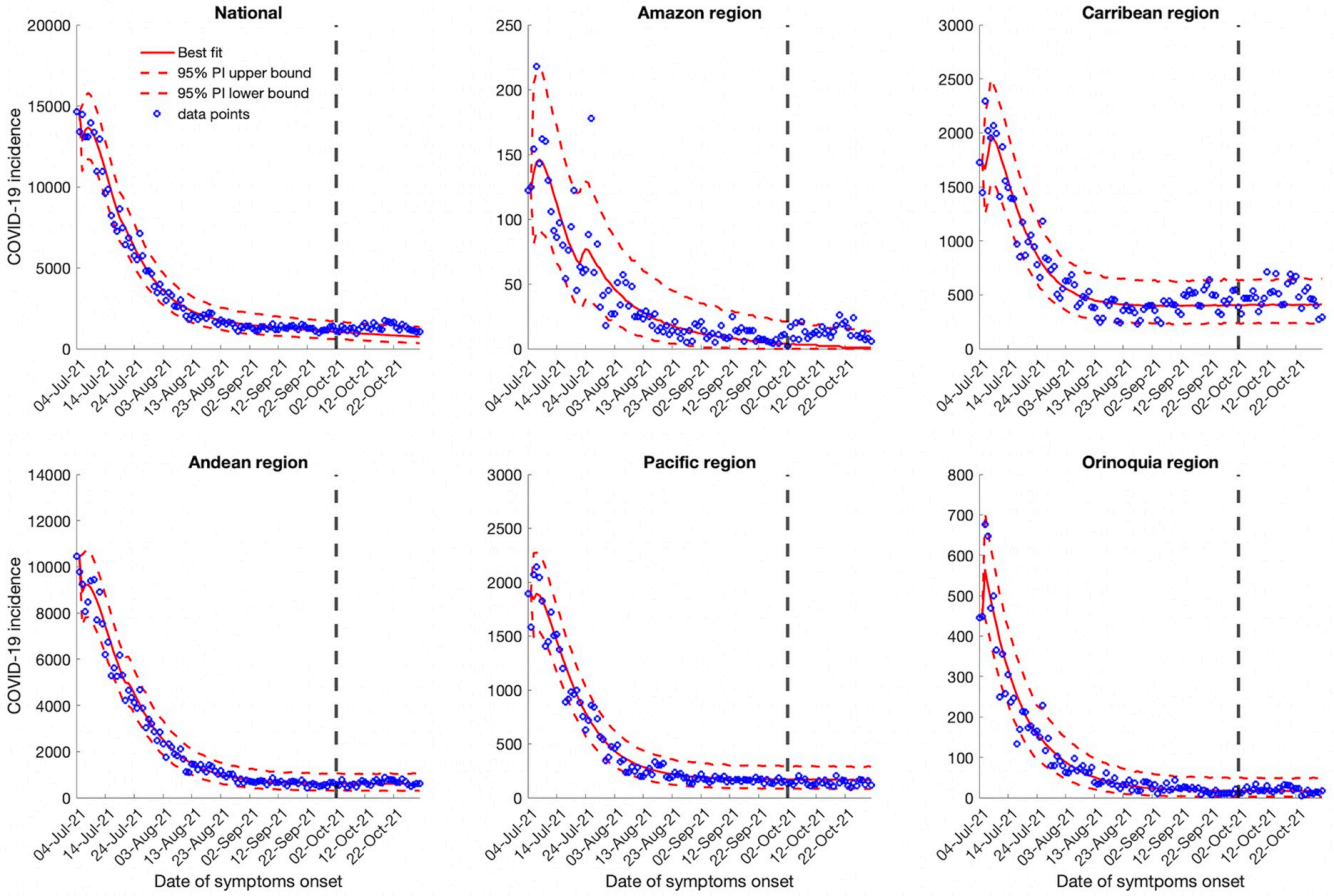
30-days ahead forecast of the national and regional COVID-19 epidemic curves in Colombia by calibrating the sub-epidemic wave model from July 4, 2021 to October 1, 2021. The blue circles correspond to the data points; the solid red line indicates the best model fit, and the red dashed lines represent the 95% prediction interval. The vertical black dashed line represents the time of the start of the forecast period.

Calibrating our models from July 04, 2021, to October 1, 2021 and generating the 30-day ahead forecasts from October 2, 2021 to October 31, 2021 utilizing the GLM and Richards growth model indicates a downwards trend for the national and regional case incidence data (Figs 3 and 4). On the other hand, the sub-epidemic wave model captures the multiple sub-epidemics comprising the coarse aggregated COVID-19 epidemic wave of Colombia. The sub-epidemic model predicts a downward trend for the Amazon and Orinoquía region, consistent with the findings of the GLM and Richards model. Whereas, for the national, Andean, Caribbean and the Pacific region, the sub-epidemic model predicts a stable case incidence pattern (Fig 5). According to the GLM and Richards model, the COVID-19 pandemic in Colombia would decline to zero during the month of October whereas the sub-epidemic wave model predicts an accumulation of 24,525 (95% PI: 13,677, 44,515) cases at the national level for the month of October 2021.

At the regional level, the Richards and the GLM model predict zero cases for the month of October (Figs 3 and 4). However, the sub-epidemic model predicts 59 (95% PI: 0, 507) cases for the Amazon region in the month of October. The sub-epidemic model also predicts 12,186 (95% PI: 7107, 19,235) cases for the Caribbean region, 18,165 (95% PI: 9205, 31,205) cases for the Andean region, 5039 (95% PI: 2517, 8725) cases for the Pacific region and 510 (95% PI: 87, 1456) cases for the Orinoquía region (Fig 5). The 30-day ahead forecast of the national mortality data generated by the GLM and Richards model indicate a decline in the number of deaths whereas the sub-epidemic wave model indicates an upward trend in the mortality curve with ~2893 (95% PI: 1860, 5325) deaths that can accumulate in the month of October 2021 (S3 Fig).

### Reproduction number

#### Estimate of the effective reproduction number, *R*_*t*_ from case incidence data

**Fig 6:**
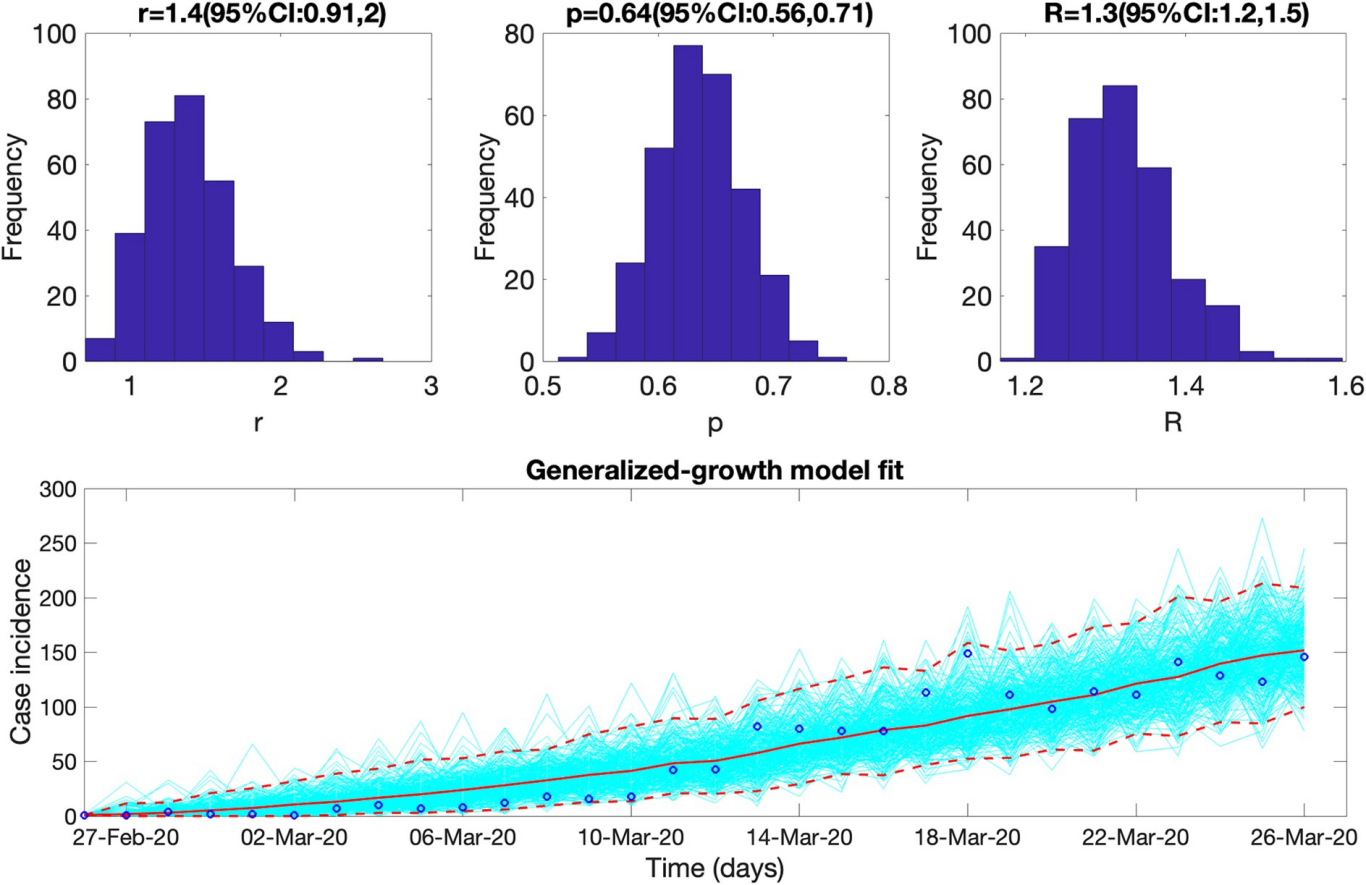
Upper panel: Reproduction number for Colombia with 95% CI estimated using the GGM model. The estimated reproduction number of the COVID-19 epidemic in Colombia as of March 27, 2020, is 1.30 (95% CI: 1.20, 1.50). The growth rate parameter, *r*, is estimated at 1.40 (95%CI: 0.91, 2.0) and the deceleration of the growth parameter, *p*, is estimated at 0.64 (95%CI: 0.56, 0.71) at *α* = 0.15. Lower panel: The lower panel shows the GGM fit to the case incidence data for the first 30 days from February 27, 2020 to March 27, 2020. The blue circles correspond to the data points; the solid red line indicates the best model fit, and the red dashed lines represent the 95% confidence interval. The cyan lines are the model fits obtained via bootstrapping.

The reproduction number for the early ascending growth phase of the epidemic from the case incidence data (February 27, 2020 to March 27, 2020) using GGM was estimated at *R*_*t*_~1.30 (95% CI:1.20, 1.50) at *α* = 0.15 for the national data. The growth rate parameter, *r*, was estimated at 1.40 (95% CI: 0.91, 2.0) and the deceleration of growth parameter, *p*, was estimated at 0.64 (95% CI: 0.56, 0.71), indicating early sub-exponential growth dynamics of the COVID-19 pandemic in Colombia (Fig 6). Simultaneously, the estimates of *R*_*t*_ for all the regions remained consistently above 1.20 (between ~1.20–2.22) for the early ascending phase of the pandemic with the Amazon region showing the highest estimate of reproduction number, at *R*_*t*_ ~2.2 followed by the Orinoquía region with *R*_*t*_~1.8. The estimates of *R*_*t*_ for the Andean, Pacific and Caribbean regions remained between *R*_*t*_~1.2–1.4. All regions except the Amazon region depict sub-exponential growth dynamics for the COVID-19 pandemic in Colombia with the deceleration of the growth parameter, *p*, estimated between 0.54–0.86. The Amazon region shows almost exponential growth dynamics with the deceleration of growth parameter, *p* ~0.95 (95% CI: 0.74, 1.00). In contrast, the Andean and the Caribbean regions show an almost linear pattern of the epidemic trajectory with the deceleration of growth parameter estimated at, *p* ~0.58 (95% CI: 0.48, 0.69) and *p* ~0.59 (95% CI: 0.34, 0.87) respectively. The sensitivity analysis shows that the estimates of reproduction numbers do not vary significantly at *α* = 0.15 and *α* = 1.00 (Tables 4 and 5). Moreover, setting *α* = 0.00, thereby assuming that there is no contribution of imported cases to the disease transmission process or generation of secondary cases also does not substantially change the estimates of reproduction number.

**Table 4:** Estimates of reproduction number (*R)*, growth rate parameter *(r)* and deceleration of the growth parameter (*p*) obtained from the renewal equation method utilizing the GGM for the early ascending phase of the epidemic (30 days) at the national and regional level at *α* = 0.15.

Region	*r*	*p*	*R*
National	1.4 (95% CI:0.91, 2.0)	0.64 (95% CI:0.56, 0.71)	1.3 (95% CI:1.2, 1.5)
Amazon	0.23 (95% CI:0.18, 0.4)	0.95 (95% CI:0.74, 1.00)	2.2 (95% CI:1.5, 2.6)
Andean	1.70 (95% CI:0.88, 2.8)	0.58 (95% CI:0.48, 0.69)	1.2 (95% CI:1.1, 1.4)
Caribbean	0.85 (95% CI:0.27, 1.9)	0.59 (95% CI:0.34, 0.87)	1.3 (95% CI:1.1,1.8)
Pacific	0.63 (95% CI:0.25, 1.3)	0.69 (95% CI:0.48, 0.90)	1.4 (95% CI:1.1, 2.0)
Orinoquía	0.25 (95% CI:0.16, 0.59)	0.87 (95% CI:0.58, 1.00)	1.8 (95% CI:1.3, 2.4)

**Table 5:** Estimates of reproduction number (*R*), growth rate parameter *(r*) and deceleration of the growth parameter (*p*) obtained from the renewal equation method utilizing the GGM for the early ascending phase of the epidemic (30 days) at the national and regional level at *α* = 1.00.

	*r*	*p*	*R*
National	1.40 (95% CI:0.87, 2.1)	0.64 (95% CI:0.56, 0.72)	1.1 (95% CI:0.97, 1.2)
Amazon	0.23 (95% CI:0.18, 0.39)	0.94 (95% CI:0.77, 1.0)	2.1 (95% CI:1.4, 2.5)
Andean	1.70 (95% CI:1.0, 2.8)	0.58 (95% CI:0.48, 0.67)	1.0 (95% CI:0.94, 1.2)
Caribbean	0.82 (95% CI:0.19, 2.00)	0.61 (95% CI:0.34, 0.99)	1.3 (95% CI:1.0, 2.3)
Pacific	0.61 (95% CI:0.23, 1.2)	0.69 (95% CI:0.51, 0.95)	1.2 (95% CI:0.93, 1.9)
Orinoquía	0.26 (95% CI:0.16, 0.53)	0.86 (95% CI:0.6, 1.0)	1.8 (95% CI:1.3, 2.4)

#### Estimate of effective (instantaneous) reproduction number, *R*_*t*_, from the Cori et al. method

**Fig 7:**
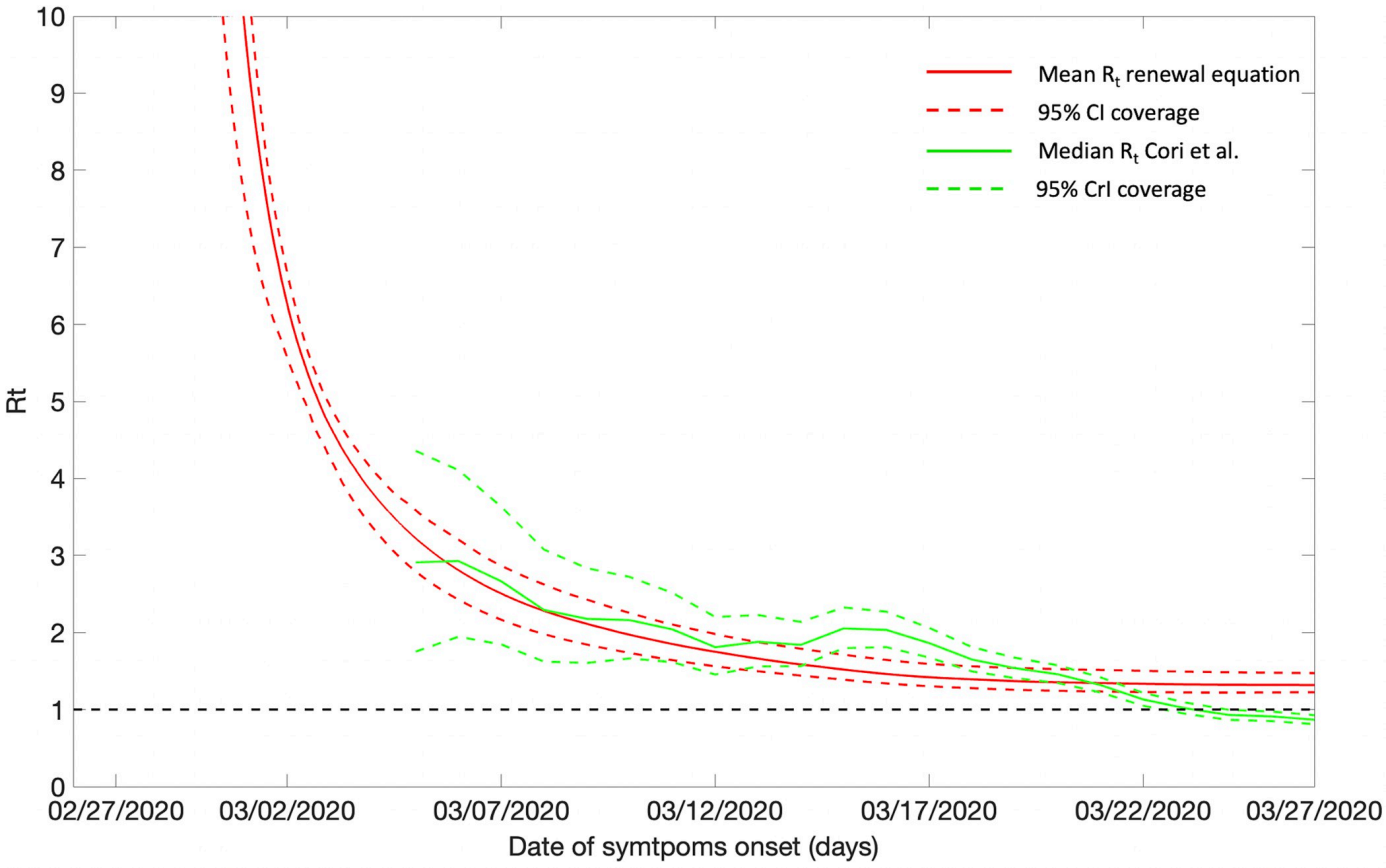
Effective (instantaneous) reproduction number with 95% CrI estimated using the Cori et al. method and the estimate of reproduction number with 95% CI utilizing the GGM for simulating the parameters employed in the renewal equation method for the COVID-19 epidemic in Colombia as of March 27, 2020 (first 30 days). The red solid line is the mean reproduction number estimated from the renewal equation method and the red dashed lines are the 95% CI coverage around the mean reproduction number. The green solid line is the median effective reproduction number estimated from the Cori et al. method and the green dashed lines are the 95% CrI coverage around the median instantaneous reproduction.

**Fig 8:**
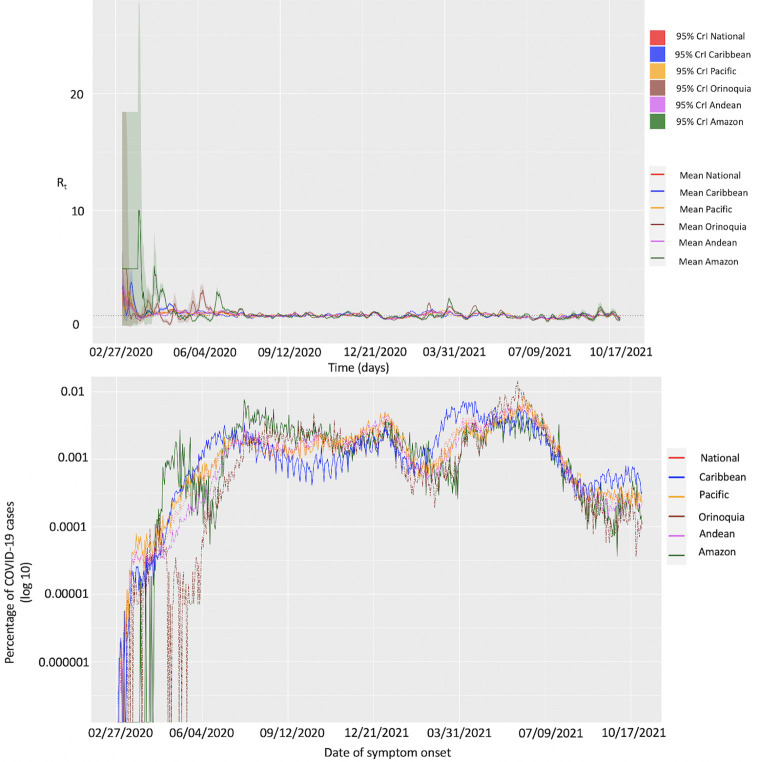
Upper panel: Effective reproduction number estimated from the Cori et al. method with 95% credible intervals for the COVID-19 pandemic in Colombia as of October 31, 2021. The red solid line represents the mean reproduction number for Colombia and the red shaded area represents the 95% credible interval around it. The blue solid line represents the mean reproduction number in the Caribbean region and the blue shaded area represents the 95% credible interval around it. The yellow solid line represents the mean reproduction number in the Pacific region and the yellow shaded area represents the 95% credible interval around it. The brown solid line represents the mean reproduction number in the Orinoquía region and the brown shaded area represents the 95% credible interval around it. The pink solid line represents the mean reproduction number in the Andean region and the pink shaded area represents the 95% credible interval around it. The green solid line represents the mean reproduction number in the Amazon and the green shaded area represents the 95% credible interval around it. Lower panel: The percentage of the COVID-19 cases in Colombia, nationally and regionally as of October 31, 2021. The red solid line represents the percentage of cases in Colombia, the blue solid line represents the percentage of cases in the Caribbean region, the yellow solid line represents the percentage of cases in the Pacific region, the brown dotted line represents the percentage of cases in the Orinoquía region, the pink dashed line represents the percentage of cases in the Andean region and the green dotted line represents the percentage of cases in the Amazon region.

The early estimate of effective (instantaneous) reproduction number at the national level for the first 30 epidemic days exhibits a steep decline. The reproduction number was estimated at, *R*_*t*_~2.85 (95% CrI: 1.91, 4.09) on March 6, 2020, which declined to *R*_*t*_~0.81 (95% CrI: 0.76, 0.85) by March 27, 2020 (Figs 7 and 8). At the national level the effective reproduction number has remained consistently above *R*_*t*_~1.00 between April and July 3, 2020, after which the reproduction number declined to less than 1.00 (*R*_*t*_~0.9) until September 22, 2020. Since the end of September 2020, *R*_*t*_ has fluctuated around ~1.00 (*R*_*t*_~ 0.8–1.4) until the end of April 2021. From May till mid-June 2021, the estimates of *R*_*t*_ remained above ~1.0 indicating continued disease transmission. Since then, the estimates of *R*_*t*_ have mostly remained below ~1.0 with the most recent estimate of *R*_*t*_~0.84 (95% CrI: 0.82, 0.86) as of October 31, 2021 (Fig 8).

At the regional level, majority of the fluctuations in *R*_*t*_ were observed during the first 130 epidemic days (until July 5, 2020) after which estimates of *R*_*t*_ remain at around ~1.0. For the Caribbean region the early estimate of reproduction number was *R*_*t*_~3.42 (95% CrI: 1.85, 5.69) on March 6, 2020. The reproduction number peaked on March 16, 2020, with an estimate of *R*_*t*_~3.46 (95% CrI: 2.69, 4.36) and declined thereafter to remain consistently above 1.00 (*R*_*t*_~1.00–3.21) until July 7, 2020. The reproduction number then fluctuated at ~1.00 until February 11, 2021. Since then, the estimates of *R*_*t*_ have remained between ~0.8–1.5 with the most recent estimate of *R*_*t*_~0.78 (95% CrI: 0.75, 0.81) as of October 31, 2020. In the Pacific region, the early estimate of reproduction number as estimated on March 5, 2020, was *R*_*t*_~3.04 (95% CrI: 1.28, 5.96). The reproduction number remained above 1.50 in the next 15 days. This was followed by the estimates of *R*_*t*_ consistently above 1.00 until the end of July 20, 2020, after which *R*_*t*_ fluctuated ~1.00 until February 2021. From March to October 31, 2021, the estimates of *R*_*t*_ remained between 0.9–1.5 with the most recent estimate of *R*_*t*_~1.01 (95% CrI: 0.09, 1.08) as of October 31, 2021. The reproduction number in the Orinoquía region was estimated at *R*_*t*_~3.46 (95% CrI: 0.25, 14.97) from March 5, 2020, to March 11, 2020, and declined thereafter to fluctuate ~1.00 until the end of May 2020. From June 1, 2020, till the end of August 2020 the estimates of *R*_*t*_ remained above 1.00 followed by fluctuations in *R*_*t*_ around 1.00. From April 9, 2021, the estimates of *R*_*t*_ have remained below 1.0 with the most recent estimate of *R*_*t*_~0.73 (95% CrI: 0.59, 0.88) as of October 31, 2021.

The reproduction number in the Andean region peaked at *R*_*t*_~3.28 (95% CrI: 2.27, 4.55) on March 6, 2020. This was followed by the estimates of reproduction number above 1.00 until July 27, 2020. Since then, *R*_*t*_ has fluctuated ~1.00 until mid-June 2021 after which the estimates of *R*_*t*_ remained consistently below 1.0 until October 10, 2021. The most recent estimate of *R*_*t*_ is at ~0.86 (95% CrI: 0.83, 0.88) as of October 31, 2021. In contrast, the reproduction number in the Amazon region was estimated at *R*_*t*_~3.46 (95% CrI: 0.25, 14.9) on March 5, 2020. This was followed by the estimates of *R*_*t*_ consistently above 1.00 until May 11, 2020 which have since fluctuated ~1.00 until July 2021. The estimates of *R*_*t*_ remained consistently below 1.00 from July 10, 2021, until September 30, 2021, with the most recent estimate of *R*_*t*_~0.64 (95% CrI: 0.07, 0.77) as of October 31, 2021 (Fig 8). The estimates of *R*_*t*_ obtained from the mortality curve remain higher than 1.00 for the first fifty days after which *R*_*t*_ fluctuates around 1.00 till October 31, 2021 (S4 Fig). As can be observed in both, the national and the regional level case incidence data and the national-level mortality data, the Cori et al. method tends to overestimate *R*_*t*_ early in the time series as infections occurring before the first date in the time series remain missing terms in the denominator [80].

#### Estimate of reproduction number, R from genomic data analysis

**Fig 9:**
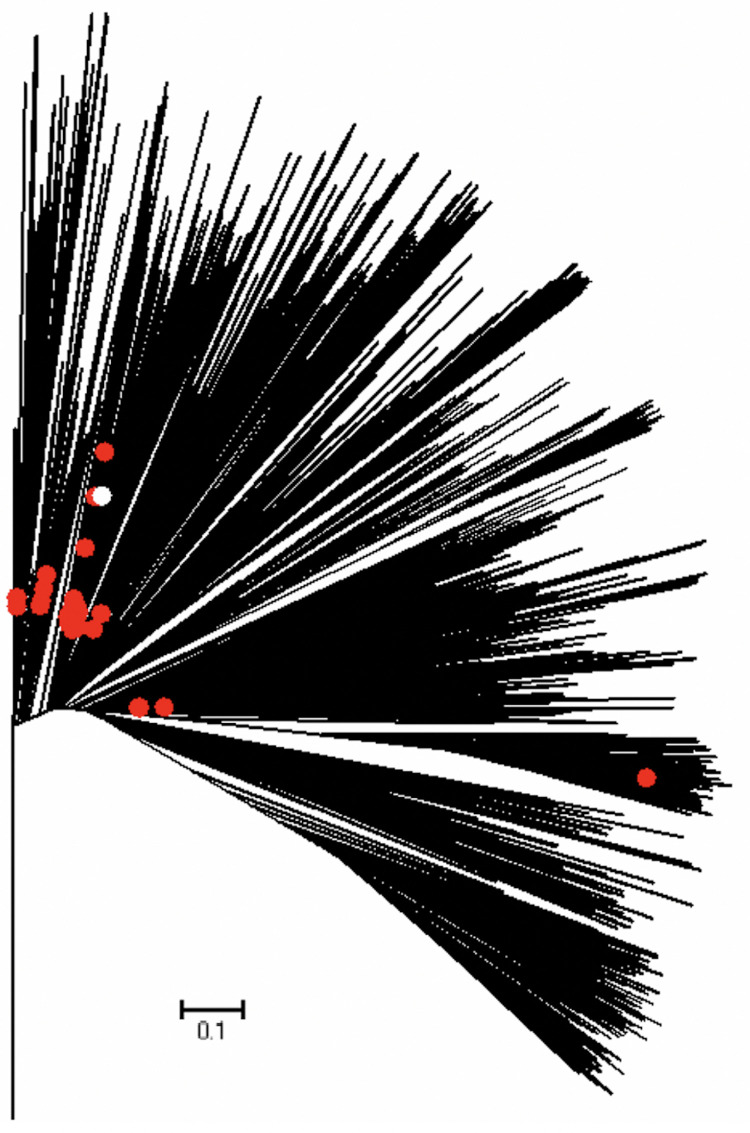
Global Maximum Likelihood (ML) tree for SARS-CoV-2 genomic data analyzed in the paper. The leaves of the tree are Colombian sequences of SARS-CoV-2 sampled between February 27, 2020, and April 5, 2020 (highlighted in red) and background sequences sampled from around the globe for genomic context. Colombian sequences are distributed among different lineages, indicating multiple virus introductions. The largest estimated intra-Colombian cluster (i.e., a monophyletic clade consisting solely of Colombian sequences) was analyzed using an exponential growth coalescent model to estimate the intra-country basic reproduction number.

Between February and April 2020, 136 analyzed Colombian sequences were sampled. These sequences were spread along the whole global SARS-CoV-2 phylogeny (Fig 9) and split into multiple clusters. This indicates multiple introductions of SARS-CoV-2 to the country during the initial pandemic stage (February 27, 2020 to April 5, 2020). For the largest cluster of size 35, growth rate for the early ascending phase of the COVID-19 pandemic in Colombia was estimated to be ~12.718. Subsequently, the reproduction number was estimated at, R~1.2 (95% HPD interval:[1.03,1.44]) indicating sustained disease transmission during the initial phase of the pandemic.

#### Spatial analysis

**Fig 10:**
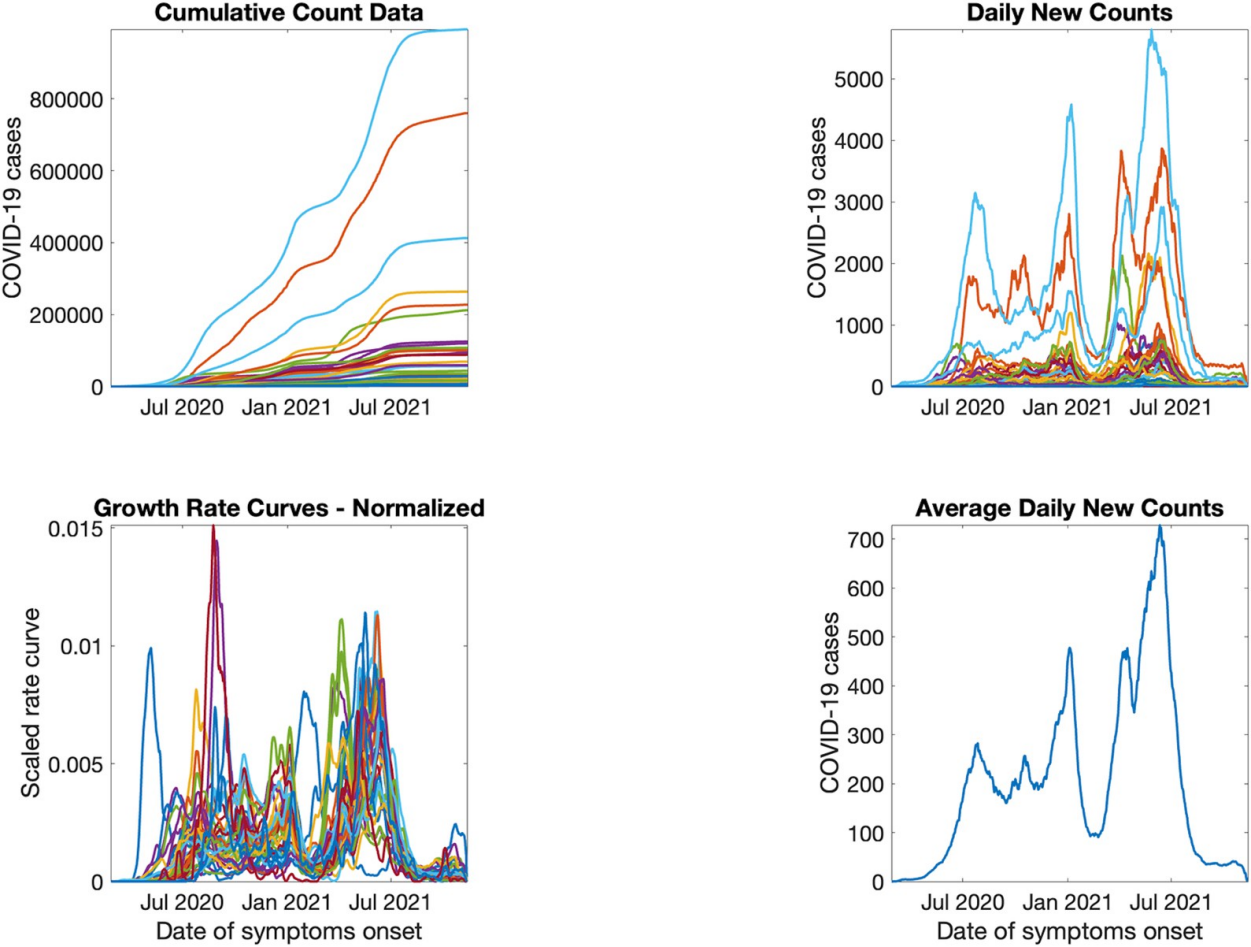
Pre-processing COVID-19 data into incidence rate functions. From left to right: the original lab-confirmed COVID-19 cases, curve of daily new cases, smoothed and scaled rate curves, and average of rate curves before scaling and smothing.

**Fig 11:**
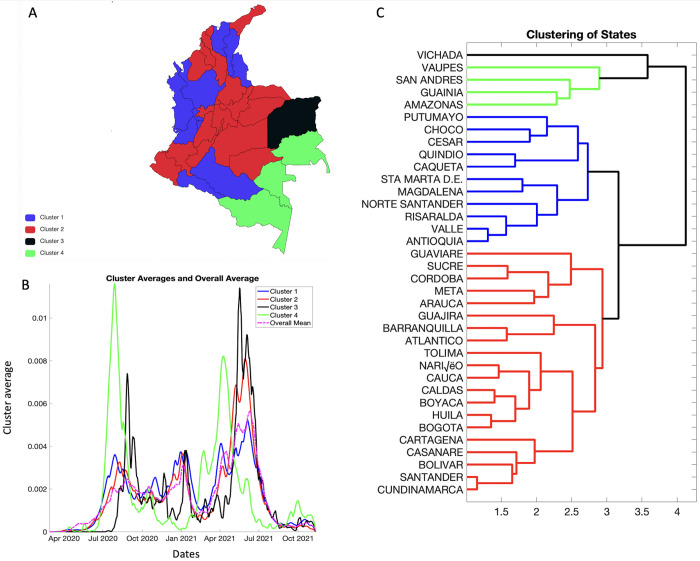
Panel A. Clustering of states according to the shapes of their rate curves. Cluster 1 is shown in blue, cluster 2 is shown in red, the smallest cluster, cluster 3 is shown in black whereas cluster 4 is shown in green. One can see that states with similar shapes of rates curves are geographically close to each other. The panel shows the geographic distribution of the clusters created in PaintMaps.com, https://paintmaps.com/map-charts/51/Colombia-map-chart [81]. Panel B. Shows the average shape of the incidence rate curves in each cluster and the overall average. Panel C. Shows the dendrogram plot- a hierarchal clustering of states, which shows that there are four predominant clusters of states.

Fig 10 shows the result from pre-processing COVID-19 data into growth rate functions. The results of clustering are shown in Fig 11. The four predominant clusters identified include the following departments and districts:

Cluster 1: Antioquia, Caquetá, Cesar, Choco, Magdalena, Norte Santander, Putumayo, Quindío, Risaralda, Sta Marta D.E., Valle del Cauca
Cluster 2: Arauca, Atlántico, Barranquilla, Bogotá, Bolívar, Boyacá, Caldas, Cartagena, Casanare, Cauca, Córdoba, Cundinamarca, La Guajira, Guaviare, Huila, Meta, Nariño, Santander, Sucre, and Tolima
Cluster 3: Vichada
Cluster 4: Amazonas, Guainía, San Andrés, and Vaupés

**Fig 12:**
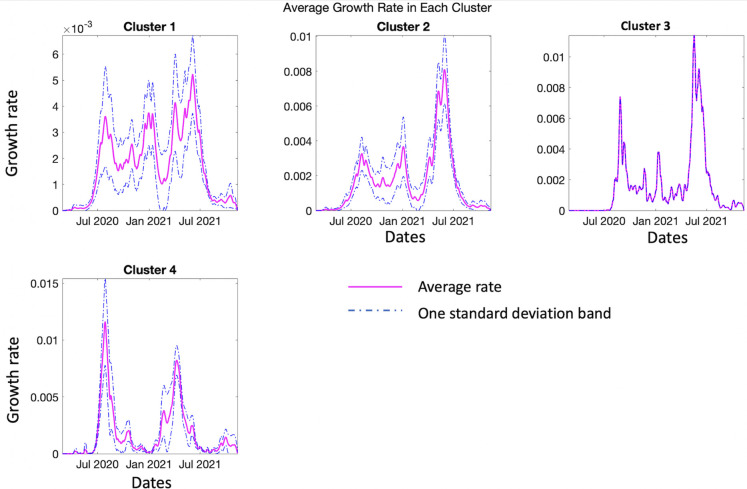
Average shapes of the COVID-19 incidence rate curves, along with a one standard-deviation band around the average, in each of the clusters.

Fig 12 shows the mean growth rate curves and one standard-deviation bands around it, in each cluster. The average incidence patterns in these four clusters are very distinct and clearly visible (Fig 12). For cluster 1, the rate increases rapidly from April 2020 to July 2020 and then declines to increase again at the end of November 2020 followed by another incline at the start of January 2021. The most recent upsurge in growth rate can be observed by the two-peak shape from May-July 2021. For cluster 2, we can observe a three modal growth pattern. There is rapid increase in growth rate in July 2020 followed by a decline and continuous fluctuations. This pattern is followed by two more peaks, one in January 2021 and the other peak can be observed in March and April 2021. Cluster 3 shows a slow growth rate until July 2020 followed by a rapid rise in growth curve until mid-September 2020. This is followed by a gradual decline of the growth curve with fluctuations and another large peak between June to July 2021. For cluster 4, the slow growth rate is followed by a spike in September 2020 and multiple fluctuations. This is followed by another peak in June 2021 (Fig 12).

### Mobility data

**Fig 13:**
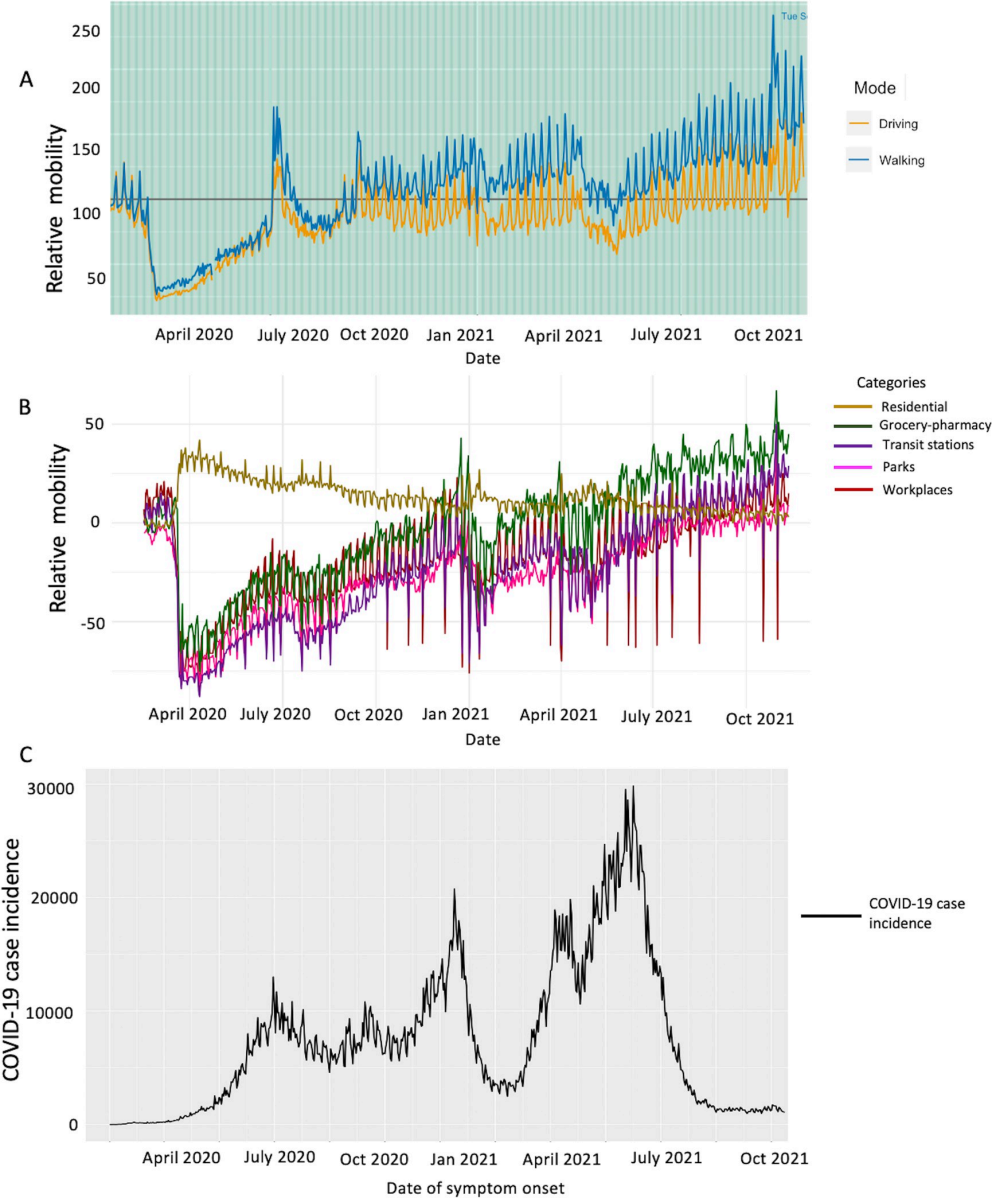
Panel A: Apple mobility trends data showing the variation in walking and driving among the population in Colombia. Orange curve shows the driving trend, and the blue curve shows the walking trend. Panel B: Google mobility trends data showing the variation in movement of people in Colombia across the following categories: grocery and pharmacy (green solid curve), parks (pink solid curve), transit stations (purple solid curve), workplaces (red solid curve) and residential areas (golden solid curve). Panel C: The COVID-19 incidence curve in Colombia by the dates of symptoms onset as of October 31, 2021.

The curve of mobility trends from Apple tracked in the form of driving and walking declined in April 2020 to less than 60% corresponding to multiple interventions focused on the social distancing mandates as implemented by the government of Colombia. Mobility then started to increase in June, peaking in July 2020. Soon after, a decline in mobility was observed in August and September 2020 which was followed by fluctuations in mobility trends around the baseline until the end of June 2021. Thereafter, fluctuations in mobility can be seen trending away from the baseline until October 31, 2021 (Fig 13). Correspondingly it can be observed that as the mobility increased in July 2020, the number of cases also peaked.

The curves of mobility from Google data tracked in the form of visits to grocery and pharmacy, parks, transit stations and workplaces all follow the same pattern; declining in April 2020 to less than 60% incoherence with the Apple mobility data and corresponding to the government’s interventions to contain the spread of the virus. The mobility started to increase gradually thereafter but remained below the baseline until December 2020. The greatest fluctuations in mobility were observed in January 2021, the same time when the cases also peaked. Mobility has since fluctuated around the baseline with an inclining trend in the mobility for transit stations and grocery and pharmacy. The residential category is measured in duration rather than the number of visitors, thus we do not compare it with other five categories. Moreover, since people spend a larger amount of time at home, changes in the residential category remain inconspicuous (Fig 13B).

### Twitter data analysis: Social media trends

**Fig 14:**
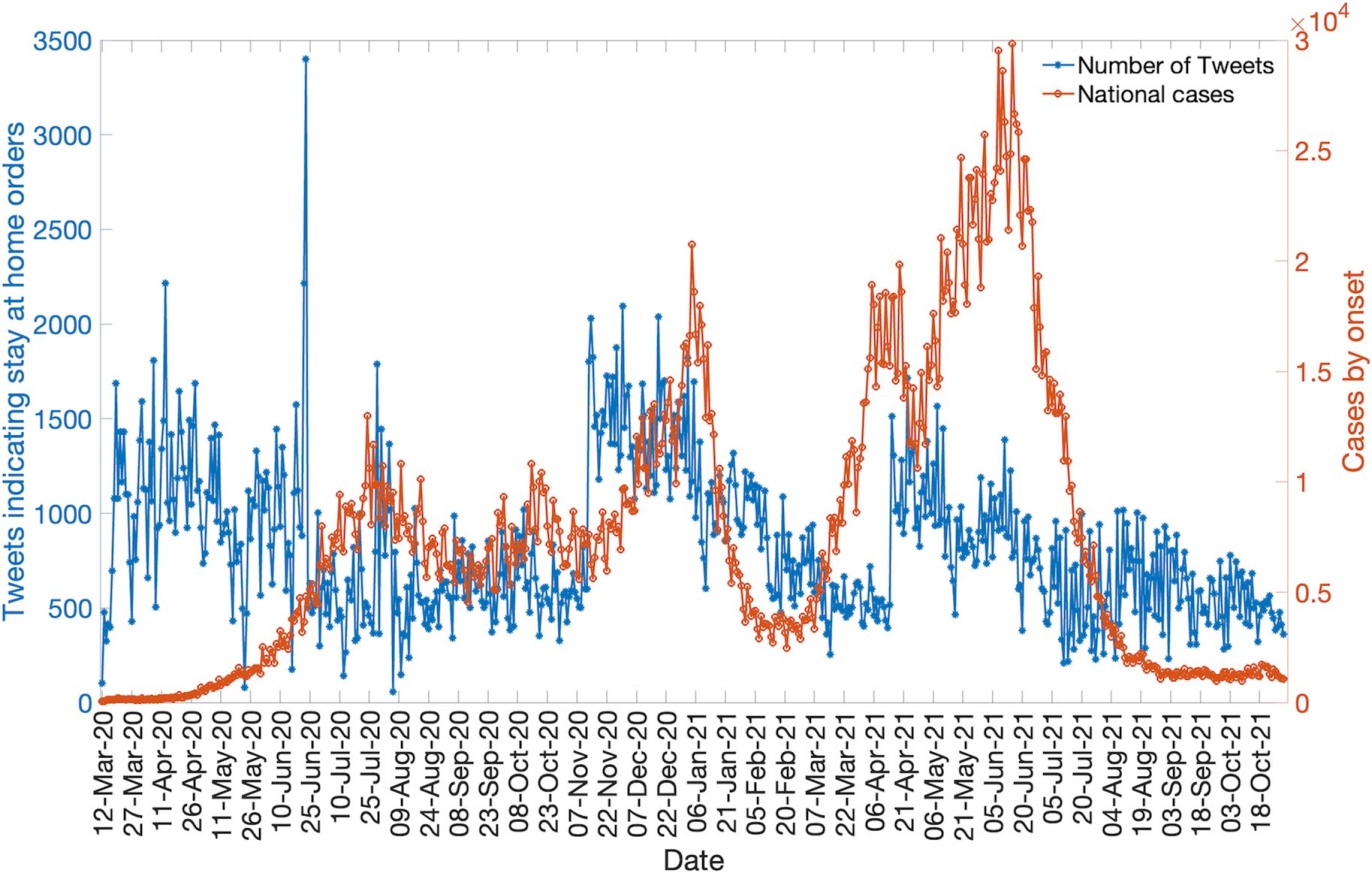
The daily number of tweets indicating stay at home orders (blue) and the daily number of new COVID-19 cases based on the dates of symptom onset (orange) as of October 31, 2021.

The epidemic curve for Colombia is overlaid with the curve of tweets indicating the stay-at-home orders in Colombia. In Colombia the engagement of people with the #quedateencasa hashtag (stay-at-home order hashtag) fluctuated at a steady level during the course of the pandemic with a slight decline in the frequency of tweets observed after June 2020 while the number of cases continued to increase, which could point towards the apathy or frustration of the public towards the lockdowns and restrictions. This could also imply that the population does not follow the government’s stay-at-home orders. The decline in the number of tweets indicating stay-at-home orders was followed by a sharp increase in the frequency of tweets in November 2020 which gradually declined until April 2021. A short spike in the frequency of tweets was observed in April 2021 which have since remained around ~500 tweets per day. On the contrary, the cases peaked in July 2020 levelling off at ~7000 cases per day until December 2020 (Fig 14). Another peak in case incidence was observed in January 2021 followed by a sharp decline. This was followed by two upsurges in case incidence in November 2020 and April 2021. The correlation coefficient between the epidemic curve of cases based on the dates of symptom onset and the curve of tweets representing the stay-at-home orders was estimated at R = 0.1797 from March 12, 2020, to October 31, 2021.

## Discussion

In this study, we characterize the forecasting efforts and spatio-temporal transmission dynamics of the COVID-19 pandemic in Colombia by fitting mathematical models to the national and spatially distributed time series data. Our results point towards the sub-epidemic model as the most accurate model in terms of the calibration and forecasting performances. More importantly, the regional and national level GLM and Richards forecasts point towards a continuous declining trend in the epidemic trajectory in comparison to the sub-epidemic model that is able to reproduce the sustained growth pattern particularly distinguishable for the national, Caribbean, Andean and the Pacific region. Overall, the transmission dynamics show sustained disease transmission during the early phase of the COVID-19 pandemic exhibiting sub-exponential growth dynamics at the regional and national levels. These findings are consistent with the estimates of reproduction number obtained from the genomic data. As the epidemic progressed, fluctuations in *R*_*t*_ were observed with the most recent estimates of *R*_*t*_<1.00 indicating disease containment. Regarding the geographical distribution in the national territory, Colombian departments can be segregated into four distinct clusters based on the growth rate patterns in each cluster. Moreover, while the increase in mobility patterns in June and July 2020 correspond to the observed peak in case incidence in July 2020, a spike in the number of tweets indicating the stay-at-home orders was observed in November 2020 when the case incidence had levelled off.

Appropriate short-term forecasts at the national and regional levels can help guide the intensity and magnitude of public health interventions required to contain the epidemic. In general, the national and regional short-term forecasts from the Richards model and the GLM point towards a sustained decline in the over-all case counts similar to the forecast produced by the sub-epidemic wave model for the Amazon and Orinoquía region. However, the sub-epidemic model predicts the stabilization in case incidence for the national, Andean, Caribbean, and the Pacific region. On the contrary, the mortality curve forecast predicted by the sub-epidemic wave model shows an increase in the death counts as is projected by the IHME model [82]. In general, the forecasts generated by the GLM, and Richards model predict that national and regional outbreaks have nearly reached extinction (Figs 3 and 4) in comparison to the forecasts generated by the sub-epidemic wave model. Therefore, the projections made using these models should be interpreted with caution given the instability in the reporting patterns and reporting delays. It can be appreciated that the sub-epidemic wave model is able to capture the underlying structure of the sub-epidemic building blocks comprising the coarse scale epidemic [46]. In our analysis the sub-epidemic wave model performs better than the GLM and Richards model in short term forecasts based on the performance metrics (Tables 3 and S2) that account for uncertainty in predictions similar to the findings in Mexico where the sub-epidemic wave model outperformed the GLM and Richards model in short term forecasting the COVID-19 pandemic [49]. The sub-epidemic wave model is also a better fit to the epidemic trajectories during the calibration period compared to the other two models (Tables 2 and S1).

The scaled COVID-19 case incidence based on the dates of symptom onset for all regions and the national data shows a coherent pattern with fluctuations in the percentage of cases around ~0.001 between April 2020 and October 2021 (Fig 8). The epidemic curve of the COVID-19 pandemic displays multimodal peaks as of October 31, 2021. As observed the COVID-19 epidemic peaked in mid-July 2020 followed by a decline in case incidence in mid-September 2020, only to rise again. The national COVID-19 curve declined at the beginning of year 2021. However, by the end of April 2021 another surge in the COVID-19 cases was observed that continued until the end of June 2021, with estimates of *R*_*t*_ ~1.00, indicating continued virus transmission in the country.

The early transmission dynamics of SARS-CoV-2 exhibit similarity at the national and regional levels. The COVID-19 pandemic in Colombia exhibited sub-exponential growth dynamics (0<*p*<1.00) during the early ascending phase of the outbreak at the national and regional levels. The sub-exponential growth pattern of the COVID-19 pandemic in Colombia can be attributed to a myriad of factors including non-homogenous mixing, spatial structure, population mobility, behavior changes and control interventions such as mask mandates and social distancing to contain the spread of the virus [83]. The results of this study are consistent with the sub-exponential growth patterns of the COVID-19 outbreaks observed in other Latin American countries including Mexico [84] and Chile [39] which also implemented mask mandates and social distancing interventions along with restricting mobility during the early growth phase of the pandemic. Simultaneously, the estimates of early transmission potential (*R*_*t*_) indicate sustained disease transmission in Colombia at the national and regional levels with *R*_*t*_>1.00. The results of our genomic analysis for Colombia agree with the sustained disease transmission in the country during the early ascending phase of the pandemic with R>1.00. These estimates indicate that although containment strategies were implemented during the first thirty days to mitigate the impact of the pandemic (Fig 1), additional interventions should be prioritized such as compulsory social distancing and intensified case surveillance. The results of our analysis are compatible with the estimates of early reproduction numbers retrieved from other countries, including Peru, Chile, Brazil and Mexico which followed similar COVID-19 outbreaks around the same time period [39,49,50,85].

We also employ the Cori et al. method to estimate the overall transmission potential (effective reproduction number) throughout the COVID-19 pandemic in Colombia to observe the spatiotemporal variation in R_t_ across different time points. Indeed multiple factors can be attributed to influence the reproduction number including the transmissibility of an infectious agent, individual contact rates, individual susceptibility and mitigation strategies [57,86]. Our results indicate that the national reproduction number remained substantially high (*R*_*t*_>1) until the end of July 2020, after which it declined and fluctuated around ~1.0 with no major deviations. The most recent estimates of *R*_*t*_ indicate a decrease in the transmission potential of the virus with *R*_*t*_ estimated at ~0.8, indicating disease containment. At the regional level, Orinoquía, Pacific and Caribbean regions displayed the greatest fluctuation in the transmission potential. Moreover, the estimates of *R*_*t*_ remained between 3.02–3.46 until March 6, 2020, for all regions. As the pandemic progressed and control interventions were put in place, the estimates of *R*_*t*_ declined to fluctuate at around ~1.0. The estimates of *R*_*t*_ remained consistently above 1.00 in the Caribbean, Andean and the Pacific regions until July 2020, after which *R*_*t*_ fluctuated at ~1.00. The most recent estimates of transmission potential, as of October 31, 2021, at the regional level show that the control interventions have a positive impact on the control of the spread of the virus.

In general, Colombia has observed sustained disease transmission of SARS-CoV-2 despite the implementation of social distancing interventions. As can also be seen from Twitter analysis, the number of cases based on the dates of onset are positively correlated with the tweets indicating the stay-at-home orders. A plausible explanation of this phenomenon could be that the people might have stopped following the government’s preventive orders to stay at home as a result of pandemic fatigue or due to confusion regarding the national and regional policies. There was a decline in the mobility profile in Colombia after the implementation of social distancing mandates and declaration of national emergency. However, as the pandemic progressed, the mobility of the public reached a normal level with fluctuations around the baseline. A study reported that the average mobility reduction in Colombia fluctuated during the pandemic, starting at ~80% in April 2020, and reaching ~30% by the end of September 2020 [87]. In comparison Argentina and Chile have reported the most steady mobility reductions of ~80% and 70% respectively during the course of the pandemic [87]. The most recent data shows an increase in mobility, although the case counts seem to be stabilizing at ~1307 cases per day for the month of October 2021.

Analyzing the data at a finer scale provides an opportunity to characterize the growth patterns and appreciate the spatially clustered pattern of COVID-19 that emerges at the departmental level. Our classification of epidemic patterns in Colombia at the department level, including the 4 main districts, shows distinct variations in growth rates across different departments. For instance, cluster 1 including Antioquia, Caquetá, Cesar, Choco, Magdalena, Norte Santander, Putumayo, Quindío, Risaralda, Sta Marta D.E., and Valle del Cauca shows a fluctuating yet multipeak rising pattern in growth rate. In contrast, cluster 3 that includes Vichada and cluster 4 that includes Amazonas, Guainía, and Vaupés show stable growth at lower rates. This information can be utilized by the departments, districts, and regions to guide their decision regarding the implementation of public health measures. For example, departments in cluster 1 show fluctuating increasing growth pattern during the first three months and may need strict public health measures to contain the epidemic compared to cluster 3 which displays slow growth during the first three months. Moreover, as the mobility increased in June and July 2020, an increase in the growth rate patterns can also be observed in cluster 1, 2 and 4 (Figs 12 and 13).

This study has some limitations. First, we excluded the last 11-day case counts from our study in to overcome the influence of reporting delays on our analysis. Specifically, we utilize the case counts based on the dates of onset in this study, because delays in case reporting, testing rates and factors related to the surveillance systems can influence our epidemic projections. Secondly, we relied on the daily updates of cases in the official surveillance system of Colombia, which can sometimes underreport the cases. Third, the phenomenological models applied in this study do not explicitly account for behavioral changes, and thus the results such as the predicted decline or stability in the epidemic trajectory should be interpreted with caution. Fourth, some authors [80] shifted the dates of symptom onset backwards to approximate the date of infection when applying the Cori et al. method to estimate the instantaneous reproduction number. However, in this study we stick to the date of symptoms onset for the sake of comparison across methods to estimate the reproduction number. Lastly, the unpredictable social component of the epidemic on ground was also a limiting factor for the study as we did not know the ground truth epidemic pattern when the forecasts were generated.

In conclusion, Colombia observed a surge in case counts as the mobility increased in July 2020; however, since the end of 2020, the national and regional reproduction numbers have fluctuated around ~1.00 indicating sustained virus transmission in the region. The most recent estimates of *R*_*t*_<1.00 indicate disease containment as of October 31, 2021. Moreover, the population of Colombia has had mixed reactions towards the stay-at-home orders as can be inferred from the Twitter analysis, contributing to the virus transmission in the country. Importantly, the spatial analysis indicates that departments like Antioquia, Caquetá, Cesar, and Choco need stronger public health strategies to contain the rising patterns in growth rates. Although Richards model and GLM point towards a sustained decline in the case and death counts in general, the sub-epidemic model indicates an upward trend in death counts and a stable case incidence pattern for the national, Pacific, Caribbean, and Andean regions. Hence, the forecasts need to be interpreted with caution given the spatial heterogeneity in transmission rates as well as dynamic implementation and lifting of the social distancing measures. The phenomenological growth models employed in this study to forecast and estimate reproduction numbers are particularly valuable for providing rapid predictions of the epidemics in complex scenarios that can be used for real-time preparedness because these models do not require specific disease transmission processes to account for the interventions.

## Supporting information

S1 Text Scaled incidence curve. (PDF)

S2 Text Model descriptions. (PDF)

S3 Text Model calibration and forecasting approach. (PDF)

S4 Text Performance metrics. (PDF)

S1 Fig Map of the geographical distribution of departments and the five Colombian regions: the Amazon, the Andean, the Orinoquía, the Caribbean, and the Pacific, edited by the author, scale:1:10000000 with QGIS.org 2020. QGIS Geographic Information System. QGIS Association. http://www.qgis.org. Using the Colombian department’s division shapefile obtained from https://datosabiertos.esri.co/datasets/colombia-covid19-coronavirus-departamento/explore?location=4.621900%2C-74.297150%2C5.62 (Open Source information provided by the National Institute of Health of Colombia, https://www.ins.gov.co. Publication date: April 1, 2020).
(TIFF)

S2 Fig National mortality curve of COVID-19 for Colombia as of October 31, 2021. (TIF)

S3 Fig 30-days ahead forecast of the national COVID-19 mortality curve in Colombia by calibrating the Richards, GLM and sub-epidemic wave model from July 04, 2021 to October 01, 2021. Blue circles correspond to the data points; the solid red line indicates the best model fit, and the red dashed lines represent the 95% prediction interval. The vertical black dashed line represents the time of the start of the forecast period.
(TIF)

S4 Fig Instantaneous reproduction number with 95% credible intervals for the COVID-19 mortality curve in Colombia as of October 31, 2021. The black solid line represents the mean reproduction number for Colombia and the black shaded area represents the 95% credible interval around it.
(TIF)

S1 Table Comparison of model performance metrics by calibrating the GLM, Richards and the sub-epidemic model for 90 days of mortality data (July 4, 2021 to October 1, 2021). Higher 95% PI coverage and lower RMSE, MAE, WIS and MIS represent better performance. Best performing model is given in bold with the superscript "a”.
(XLSX)

S2 Table Comparison of 30-day ahead forecasting performance (October 2, 2021 to October 31, 2021) by calibrating the GLM, Richards and the sub-epidemic model for 90 days of mortality data (July 4, 2021 to October 1, 2021). Higher 95% PI coverage and lower RMSE, MAE, WIS and MIS represent better performance. Best performing model is given in bold with the superscript "a”.
(XLSX)
