# Supplementary material for: An investigation of spatial-temporal patterns and predictions of the coronavirus 2019 pandemic in Colombia, 2020–2021
Source: PLoS Negl Trop Dis. 2022 Mar 4;16(3):e0010228. doi: 10.1371/journal.pntd.0010228 (PMC8926206; doi:10.1371/journal.pntd.0010228)
Supplement: S1 Text — (PDF) [file pntd.0010228.s001.pdf]

An investigation of spatial-temporal patterns and predictions of the coronavirus 2019 pandemic in Colombia, 2020-2021

Online Supplementary Materials

Amna Tariq*[1], Tsira Chakhaia[1] , Sushma Dahal[1], Alexander Ewing[1], Xinyi Hua[2] , Sylvia K. Ofori[2] , Olaseni Prince[1], Argita D. Salindri[1] , Ayotomiwa Ezekiel Adeniyi[3] , Juan M. Banda[3], Pavel Skums[3], Ruiyan Luo[1], Leidy Y. Lara Díaz[4], Raimund Bürger[4], Isaac Chun-Hai Fung[2], Eunha Shim[5], Alexander Kirpich[1], Anuj Srivastava[6], Gerardo Chowell[1]

[1] Department of Population Health Sciences, School of Public Health, Georgia State University, Atlanta, GA, USA

[2] Department of Biostatistics, Epidemiology and Environmental Health Sciences, Jiann-Ping Hsu College of Public Health, Georgia Southern University, Statesboro, GA, USA

[3] Department of Computer Science, College of Arts and Sciences, Georgia State University, Atlanta, GA, USA

[4] Centro de Investigación en Ingeniería Matemática (CI²MA) and Departamento de Ingeniería Matemática, Universidad de Concepción, Concepción, Chile

[5] Department of Mathematics, Soongsil University, 369 Sangdoro, Dongjak-Gu, Seoul, 06978,

Republic of Korea

[6] Department of Statistics, Florida State University, Tallahassee, Florida, USA

*atariq1@student.gsu.edu

S1. Scaled incidence curve

We scale the daily case incidence curves of COVID-19 for the regional and national level by dividing the daily number of COVID-19 cases to the total number of cases reported at the national or regional level as of October 31, 2021.
