# Supplementary material for: An investigation of spatial-temporal patterns and predictions of the coronavirus 2019 pandemic in Colombia, 2020–2021
Source: PLoS Negl Trop Dis. 2022 Mar 4;16(3):e0010228. doi: 10.1371/journal.pntd.0010228 (PMC8926206; doi:10.1371/journal.pntd.0010228)
Supplement: S2 Text — (PDF) [file pntd.0010228.s002.pdf]

An investigation of spatial-temporal patterns and predictions of the coronavirus 2019 pandemic in Colombia, 2020-2021

Online Supplementary Materials

Amna Tariq*[1], Tsira Chakhaia[1] , Sushma Dahal[1], Alexander Ewing[1], Xinyi Hua[2] , Sylvia K. Ofori[2] , Olaseni Prince[1], Argita D. Salindri[1] , Ayotomiwa Ezekiel Adeniyi[3] , Juan M. Banda[3], Pavel Skums[3], Ruiyan Luo[1], Leidy Y. Lara Díaz[4], Raimund Bürger[4], Isaac Chun-Hai Fung[2], Eunha Shim[5], Alexander Kirpich[1], Anuj Srivastava[6], Gerardo Chowell[1]

[1] Department of Population Health Sciences, School of Public Health, Georgia State University, Atlanta, GA, USA

[2] Department of Biostatistics, Epidemiology and Environmental Health Sciences, Jiann-Ping Hsu College of Public Health, Georgia Southern University, Statesboro, GA, USA

[3] Department of Computer Science, College of Arts and Sciences, Georgia State University, Atlanta, GA, USA

[4] Centro de Investigación en Ingeniería Matemática (CI²MA) and Departamento de Ingeniería Matemática, Universidad de Concepción, Concepción, Chile

[5] Department of Mathematics, Soongsil University, 369 Sangdoro, Dongjak-Gu, Seoul, 06978,

Republic of Korea

[6] Department of Statistics, Florida State University, Tallahassee, Florida, USA

*atariq1@student.gsu.edu

S2. Model descriptions

(i) Generalized logistic growth model

The generalized logistic growth model (GLM) [1] displays a range of epidemic growth patterns including the polynomial and exponential growth patterns. GLM characterizes epidemic growth by estimating three parameters: (i) the intrinsic growth rate, *r* (ii) a dimensionless "deceleration of growth" parameter, $p \in [0,1]$ and (iii) k_0, representing the final epidemic size [1]. The varied epidemic growth patterns are observed by the modulation of deceleration of growth parameter resulting in the exponential growth dynamics (*p*=1), sub-exponential growth (0<*p*<1), or constant incidence (*p*=0) patterns, if $k_0 = \infty$. When $k_0 < \infty$ and *p*=1, the GLM is the simple logistic growth model. The following differential equation gives the GLM model:

$$\frac{dC(t)}{dt} = rC(t)^p \left(1 - \frac{C(t)}{k_0}\right), \quad (1)$$

where $C(t)$ denotes the cumulative number of cases at time t, and $dC(t)/dt$ describes the incidence at time *t* [1].

(ii) Richards growth model

The well-known Richards model [2] is a simple extension of the logistic model that relies on three parameters; growth rate, r, final epidemic size, k_o and the scaling parameter, *a.* The scaling parameter, *a,* measures the deviation from the symmetric S-shaped dynamics exhibited by the simple logistic growth curve [2-4]. The following differential equation gives the Richards model:

$$\frac{dC(t)}{dt} = rC(t)\left[1 - \left(\frac{C(t)}{k_o}\right)^a\right],$$

where $C(t)$ represents the cumulative case count at time t. We remark that the Richards growth model has the explicit solution

$$C(t) = \frac{k_0 C(0) \exp(rt)}{k_0^a + C(0)^a (\exp(art) - 1))^{1/a}},$$

while the GLM does not admit a closed-form solution. For a unified treatment of all phenomenological growth models, we always refer to the corresponding differential equation in each case irrespective of the existence of a closed-form solution. Details are provided in a prior study [5].

(iii) Sub-epidemic wave model.

The sub-epidemic model [6] is based on the premise that various profiles of overlapping sub-epidemics shape the aggregated reported epidemic wave. In particular, this modeling approach supports complex temporal dynamic patterns, such as oscillating dynamics leading to damped oscillations or endemic states. This model characterizes each sub-epidemic utilizing a three-parameter generalized logistic growth model as explained above and given in equation (1). We model an epidemic wave comprising of *n* overlapping sub-epidemics using a system of coupled differential equations, as follows:

$$\frac{dC_i(t)}{dt} = rA_{i-1}(t)C_i(t)^p \left(1 - \frac{C_i(t)}{k_i}\right)$$

In this equation, $C_i(t)$ describes the cumulative cases for the ith sub-epidemic, and k_i is the size of sub-epidemic i (i=1,2,….n). Parameters r and p are the same across the sub-epidemics. The coefficient $A_i(t)$ is an indicator variable that models the onset timing of (i+1)th sub-epidemic, making sure that sub-epidemics comprising an epidemic wave follow a regular structure. Therefore,

$$A_i(t) = \begin{cases} 1 & C_i(t) > C_{thr} \\ 0 & Otherwise \end{cases} \quad i = 1,2,3, \dots n\text{-}1,$$

Where $1 \le C_{thr} < k_o$ and $A_0(t) = 1$ for the first sub-epidemic. Therefore, when n = 1 and p = 1, the sub-epidemic model becomes the simple logistic model. Moreover, for the subsequently occurring sub-epidemics, the size of ith sub-epidemic (k_i) declines exponentially at a rate q:

$$k_i = k_0 e^{-q(i-1)},$$

where k_0 is the size of the initial sub-epidemic ($k_i = k_0$). The exponential decline in the size of ith sub-epidemic can occur due to multiple factors, including the effect of interventions, changes in disease transmission dependent on seasonality and behavior changes [6].

(iv) Generalized growth model (GGM)

The generalized growth model (GGM) characterizes the early ascending phase of the epidemic by estimating two parameters: (1) the intrinsic growth rate, r; and (2) a dimensionless "deceleration of growth" parameter, p. The deceleration of growth parameter, p, allows this model to capture a range of epidemic growth profiles. The following differential equation gives the GGM model:

$$\frac{dC(t)}{dt} = C'(t) = rC(t)^p$$

In this equation, $C'(t)$ describes the incidence curve over time t, solution $C(t)$ describes the cumulative number of cases at time t and p is the modulating "deceleration of growth" parameter ($0 \le p \le 1$). This equation displays constant incidence over time if p=0 and becomes an exponential growth model for cumulative cases if p =1. The model shows sub-exponential growth dynamics if p is in the range 0< p <1 [3, 7].

References

1. Shanafelt DW, Jones G, Lima M, Perrings C, Chowell G. Forecasting the 2001 Foot-and-Mouth Disease Epidemic in the UK. EcoHealth. 2018;15(2):338-47.10.1007/s10393-017-1293-2
2. Richards FJ. A Flexible Growth Function for Empirical Use. Journal of Experimental Botany. 1959;10(2):290-301.10.1093/jxb/10.2.290
3. Chowell G. Fitting dynamic models to epidemic outbreaks with quantified uncertainty: A primer for parameter uncertainty, identifiability, and forecasts. Infectious Disease Modelling. 2017;2(3):379-98.https://doi.org/10.1016/j.idm.2017.08.001
4. Wang XS, Wu J, Yang Y. Richards model revisited: validation by and application to infection dynamics. Journal of Theoretical Biology. 2012;313:12-9.10.1016/j.jtbi.2012.07.024
5. Bürger R, Chowell G, Lara-Díaz LY. Measuring differences between phenomenological growth models applied to epidemiology. Math Biosci. 2021;334:108558.https://doi.org/10.1016/j.mbs.2021.108558
6. Chowell G, Tariq A, Hyman JM. A novel sub-epidemic modeling framework for short-term forecasting epidemic waves. BMC Med. 2019;17(1):164.10.1186/s12916-019-1406-6
7. Viboud C, Simonsen L, Chowell G. A generalized-growth model to characterize the early ascending phase of infectious disease outbreaks. Epidemics. 2016;15:27-37.https://doi.org/10.1016/j.epidem.2016.01.002
