# Supplementary material for: An investigation of spatial-temporal patterns and predictions of the coronavirus 2019 pandemic in Colombia, 2020–2021
Source: PLoS Negl Trop Dis. 2022 Mar 4;16(3):e0010228. doi: 10.1371/journal.pntd.0010228 (PMC8926206; doi:10.1371/journal.pntd.0010228)
Supplement: S3 Text — (PDF) [file pntd.0010228.s003.pdf]

An investigation of spatial-temporal patterns and predictions of the coronavirus 2019 pandemic in Colombia, 2020-2021

Online Supplementary Materials

Amna Tariq*[1], Tsira Chakhaia[1], Sushma Dahal[1], Alexander Ewing[1], Xinyi Hua[2], Sylvia K. Ofori[2], Olaseni Prince[1], Argita D. Salindri[1], Ayotomiwa Ezekiel Adeniyi[3], Juan M. Banda[3], Pavel Skums[3], Ruiyan Luo[1], Leidy Y. Lara Díaz[4], Raimund Bürger[4], Isaac Chun-Hai Fung[2], Eunha Shim[5], Alexander Kirpich[1], Anuj Srivastava[6], Gerardo Chowell[1]

[1] Department of Population Health Sciences, School of Public Health, Georgia State University, Atlanta, GA, USA

[2] Department of Biostatistics, Epidemiology and Environmental Health Sciences, Jiann-Ping Hsu College of Public Health, Georgia Southern University, Statesboro, GA, USA

[3] Department of Computer Science, College of Arts and Sciences, Georgia State University, Atlanta, GA, USA

[4] Centro de Investigación en Ingeniería Matemática (CI²MA) and Departamento de Ingeniería Matemática, Universidad de Concepción, Concepción, Chile

[5] Department of Mathematics, Soongsil University, 369 Sangdoro, Dongjak-Gu, Seoul, 06978,

Republic of Korea

[6] Department of Statistics, Florida State University, Tallahassee, Florida, USA

*atariq1@student.gsu.edu

S3. Model calibration and forecasting approach

We estimate the best-fit solution for each model using non-linear least squares fitting procedure for each of the three models (i.e., the GLM, Richards growth model and the sub-epidemic wave model) [1]. This process yields the best set of parameter estimates $\widehat{\Theta} = (\theta_1, \theta_2, \ldots, \theta_m)$, where m is the number of parameters of interest, by minimizing the sum of squared errors between the model fit, $f(t_i, \widehat{\Theta})$ and the observed data, y_{t_i}. The parameters $\widehat{\Theta} = argmin \sum_{i=1}^{n}(f(t_i, \widehat{\Theta}) - y_{t_i})^2$ define the best fit model solution, $f(t, \Theta)$, where t_i is the time stamps at which time series data are observed and n is the number of data points available for inference. In this analysis, $\widehat{\Theta} = (r, p, k_o, q \; and \; C_{thr})$ corresponds to the set of parameters of the sub-epidemic model, $\widehat{\Theta} = (r, k_0, a)$ corresponds to set of parameters of the Richards model, $\widehat{\Theta} = (r, p, k_0)$ corresponds to the set of parameters of the GLM model [2]. For the sub-epidemic wave model, we determine the initial best guesses of parameter estimates. However, for the GLM and Richards growth model we initialize the parameter estimates for the nonlinear least squares method [1] over a wide range of plausible parameters from a uniform distribution using Latin hypercube sampling [3]. This allows us to test the uniqueness of the best model fit. The initial conditions are set at the first data point for each of the three models [2].

Uncertainty bounds around the best-fit solution are generated using a parametric bootstrap approach with replacement of data, where we assume a negative binomial error structure for the sub-epidemic model. A negative binomial error structure is also used to generate the uncertainty bounds of the Richards growth model and the GLM. For both these models, using the case incidence data the variance is assumed to be 488.85 times of the mean for national data, 11.59 times of the mean for Amazon region, 356.8 times of the mean for Andean region, 69.72 times of the mean for the Caribbean region, 77.93 times the mean for the pacific region, and 22.17 times of the mean for the Orinoquia region. For the mortality data the variance is

assumed to be 17.95 times of the mean. This variance is based on the noise in the data and calculated by averaging mean to variance ratio obtained from the data. A detailed description of this method is provided in a prior study [2].

Each of the M=300 best-fit parameter sets is used to construct the 95% confidence intervals for each parameter by refitting the models to each of the M datasets generated by the bootstrap approach during the calibration phase. Further, each of the M best-fit model solutions is used to generate m= 30 additional simulations with a negative binomial error structure for the GLM, Richards and the sub-epidemic wave model extended through a 30-day forecasting period. Finally, we construct the 95% prediction intervals using the 9000 ($M \times m$) curves for the forecasting period. A detailed description of the parameter estimation methods can be found in previous literature [2, 4, 5].

References

1. Banks HT, Hu S, Thompson WC. Modeling and inverse problems in the presence of uncertainty: CRC Press; 2014.
2. Chowell G. Fitting dynamic models to epidemic outbreaks with quantified uncertainty: A primer for parameter uncertainty, identifiability, and forecasts. Infectious Disease Modelling. 2017;2(3):379-98.https://doi.org/10.1016/j.idm.2017.08.001
3. Mathworks. lhs design [cited Date].Available from: https://www.mathworks.com/help/stats/lhsdesign.html.
4. Roosa K, Luo R, Gerardo C. Comparative assessment of parameter estimation methods in the presence of overdispersion: a simulation study. Mathematical Biosciences and Engineering. 2019;16(5):4299-313
5. Roosa K, Chowell G. Assessing parameter identifiability in compartmental dynamic models using a computational approach: application to infectious disease transmission models. Theoretical Biology and Medical Modelling. 2019;16(1):1.10.1186/s12976-018-0097-6
