# Supplementary material for: An investigation of spatial-temporal patterns and predictions of the coronavirus 2019 pandemic in Colombia, 2020–2021
Source: PLoS Negl Trop Dis. 2022 Mar 4;16(3):e0010228. doi: 10.1371/journal.pntd.0010228 (PMC8926206; doi:10.1371/journal.pntd.0010228)
Supplement: S4 Text — (PDF) [file pntd.0010228.s004.pdf]

An investigation of spatial-temporal patterns and predictions of the coronavirus 2019 pandemic in Colombia, 2020-2021

Online Supplementary Materials

Amna Tariq*[1], Tsira Chakhaia[1], Sushma Dahal[1], Alexander Ewing[1], Xinyi Hua[2], Sylvia K. Ofori[2], Olaseni Prince[1], Argita D. Salindri[1], Ayotomiwa Ezekiel Adeniyi[3], Juan M. Banda[3], Pavel Skums[3], Ruiyan Luo[1], Leidy Y. Lara Díaz[4], Raimund Bürger[4], Isaac Chun-Hai Fung[2], Eunha Shim[5], Alexander Kirpich[1], Anuj Srivastava[6], Gerardo Chowell[1]

[1] Department of Population Health Sciences, School of Public Health, Georgia State University, Atlanta, GA, USA

[2] Department of Biostatistics, Epidemiology and Environmental Health Sciences, Jiann-Ping Hsu College of Public Health, Georgia Southern University, Statesboro, GA, USA

[3] Department of Computer Science, College of Arts and Sciences, Georgia State University, Atlanta, GA, USA

[4] Centro de Investigación en Ingeniería Matemática (CI²MA) and Departamento de Ingeniería Matemática, Universidad de Concepción, Concepción, Chile

[5] Department of Mathematics, Soongsil University, 369 Sangdoro, Dongjak-Gu, Seoul, 06978,

Republic of Korea

[6] Department of Statistics, Florida State University, Tallahassee, Florida, USA

*atariq1@student.gsu.edu

S4. Performance metrics

We utilize the following five performance metrics to assess the quality of our model fit and the 30-day ahead short-term forecasts: the mean absolute error (MAE) [1], the root mean squared error (RMSE) [2], the coverage of the 95% prediction intervals (95% PI) [2], the mean interval score (MIS) [2] and the weighted interval score (WIS) [2] for each of the three models: GLM, Richards model and the sub-epidemic model.

We compare the model fit to the incidence data fitted to the model for evaluating the calibration performance. In contrast, we compare our forecasts with the incidence data for the forecast period for evaluating forecast performance.

The RMSE and MAE assess the average deviations of the model fit to the observed data. The MAE is given by

$$MAE = \frac{1}{n}\sum_{i=1}^{n} |f(t_i, \widehat{\Theta}) - y_{t_i}|.$$

The RMSE is given by

$$RMSE = \sqrt{\frac{1}{n}\sum_{i=1}^{n}(f(t_i, \widehat{\Theta}) - y_{t_i})^2}.$$

In both these equations, $y_{t_i}$is the time series of cases by date of onset, t_i is the time stamp and $\widehat{\Theta}$ is the set of model parameters. For the calibration period, *n* equals the number of data points used for calibration, and for the forecasting period, *n* = 30 for the 30-day ahead short-term forecast.

Moreover, in order to assess the model uncertainty and performance of PI, we used the 95% PI and MIS. The prediction coverage is defined as the proportion of observations that fall within the 95% PI and is calculated as

$$PI\ coverage = \frac{1}{n}\sum_{i=1}^{n} I\{y_{t_i} > L_{t_i} \cap y_{t_i} < U_{t_i}\},$$

where y_{t_i} is the case incidence data, L_{t_i} and $U_{t_i}$are the lower and upper bounds of the 95% PIs, respectively, n is the length of the period, and I is an indicator variable that equals 1 if value of y_{t_i} is in the specified interval and 0 otherwise.

The MIS addresses the width of the PI as well as the coverage. The MIS is expressed as

$$MIS = \frac{1}{n}\sum_{i=1}^{n}(U_{t_i} - L_{t_i}) + \frac{2}{0.05}(L_{t_i} - y_{t_i})I\{y_{t_i} < L_{t_i}\} + \frac{2}{0.05}(y_{t_i} - U_{t_i})I\{y_{t_i} > U_{t_i}\}.$$

In this equation L_{t_i}, U_{t_i}, y_{t_i}, n and I are specified above for PI coverage. Therefore, if the PI coverage is 1, the MIS is the average width of the interval across each time point. For two models with equivalent PI coverage, a lower MIS value indicates narrower intervals [2].

Weighted interval score

Weighted interval score (WIS) is a proper score that provides quantiles of predictive forecast distribution by combining a set of interval scores (IS) for probabilistic forecasts. An interval score is simple proper score requires only a central (1−α)×100% prediction interval (PI) [2] and is described as

$$IS_{\alpha}(F, y) = (u - l) + \frac{2}{\alpha} \times (l - y)I(y < 1) + \frac{2}{\alpha}(y - u)I(y > u)$$

In this equation I refers to the indicator function, meaning that $I(y < l) = 1$ if $y < l$ and 0 otherwise. The terms l and u represent the $\frac{\alpha}{2}$ and $1 - \frac{\alpha}{2}$ quantiles of F. The IS consists of three distinct quantities:

1. The sharpness of F, given by the width $u - l$ of the central $(1 - \alpha)$PI.
2. A penalty term $\frac{2}{\alpha} \times (l - y) \times I(y < l)$ for the observations that fall below the lower end point l of the $(1 - \alpha) \times 100\%$ PI. This penalty term is directly proportional to the distance between the lower end l of the PI and y. The strength of the penalty depends on the level α.
3. An analogous penalty term $\frac{2}{\alpha} \times (y - u) \times I(y > u)$ for all the observations that fall above the upper end u of the PI.

To provide more detailed and accurate information on the entire predictive distribution, we report several central PIs at different intervals $(1 - \alpha_1) < (1 - \alpha_2) < \cdots < (1 - \alpha_k)$ along with the predictive median, m, which can be seen as a central prediction interval at level $1 - \alpha_0 \longrightarrow 0$. This is referred to as the WIS and it can be evaluated as follows:

$$WIS_{\alpha_{0:K}}(F, y) = \frac{1}{K + \frac{1}{2}} . (w_0 . |y - m| + \sum_{k=1}^{K} w_k . IS_{\alpha_k}(F, y))$$

Where, $w_k = \frac{\alpha_k}{2}$ for $k = 1,2, \ldots . K$ and $w_0 = \frac{1}{2}$. Hence, WIS can be interpreted as a measure of how close the entire distribution is to the observation, in units on the scale of the observed data [3, 4].

References

1. Kuhn M, Johnson K. Applied predictive modeling. 1st ed. New York: Springer; 2013.
2. Gneiting T, Raftery AE. Strictly Proper Scoring Rules, Prediction, and Estimation. J Am Stat Assoc. 2007;102(477):359-78.10.1198/016214506000001437
3. Bracher J, Ray EL, Gneiting T, Reich NG. Evaluating epidemic forecasts in an interval format. PLoS Comp Biol. 2021;17(2):e1008618.10.1371/journal.pcbi.1008618
4. Cramer EY, Ray EL, Lopez VK, Bracher J, Brennen A, Rivadeneira AJC, et al. Evaluation of individual and ensemble probabilistic forecasts of COVID-19 mortality in the US. medRxiv. 2021:2021.02.03.21250974.10.1101/2021.02.03.21250974
