# Supplementary figures and images for: An investigation of spatial-temporal patterns and predictions of the coronavirus 2019 pandemic in Colombia, 2020–2021
Source: PLoS Negl Trop Dis. 2022 Mar 4;16(3):e0010228. doi: 10.1371/journal.pntd.0010228 (PMC8926206; doi:10.1371/journal.pntd.0010228)

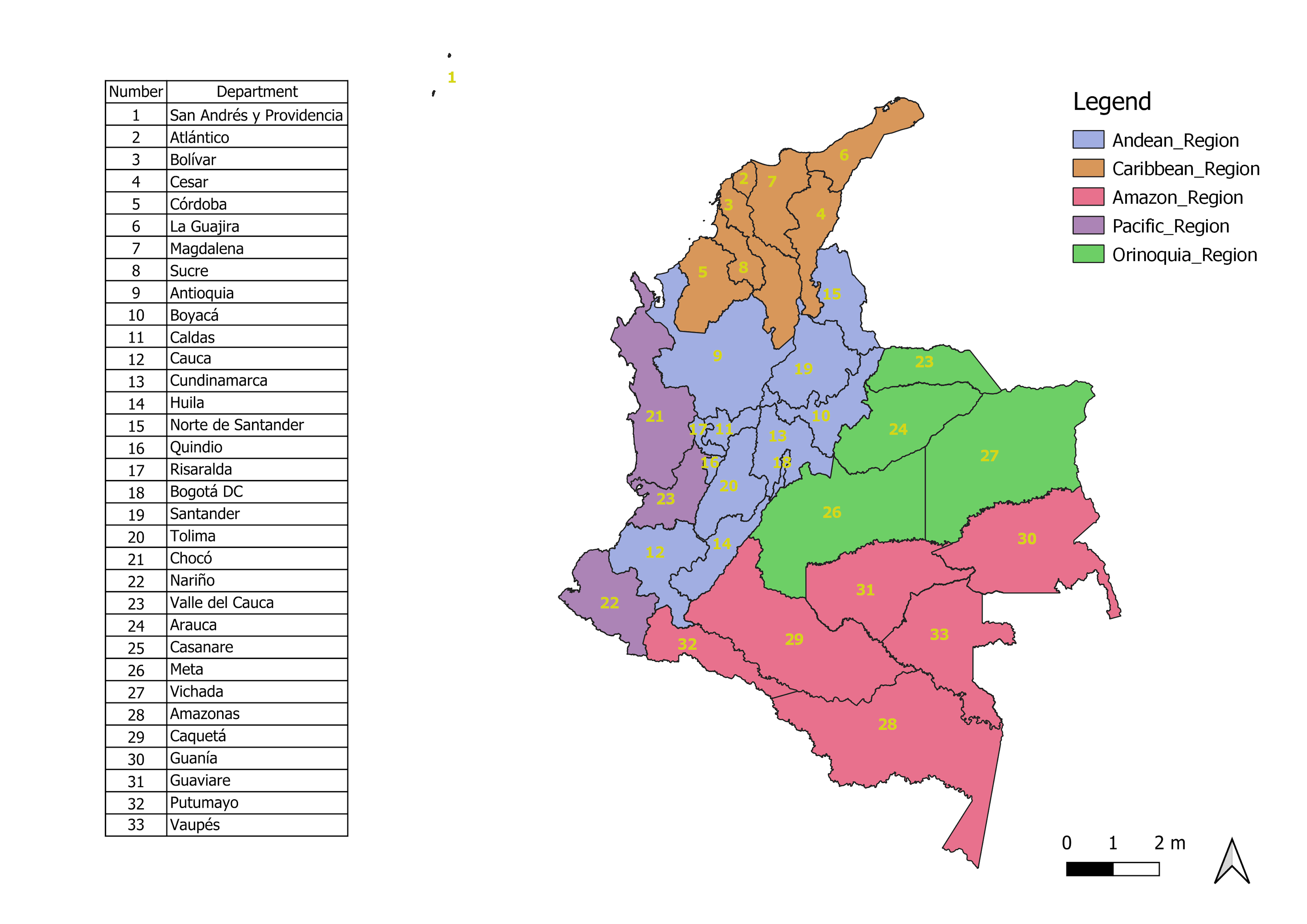

Supplement: S1 Fig — QGIS Geographic Information System. QGIS Association. http://www.qgis.org. Using the Colombian department’s division shapefile obtained from https://datosabiertos.esri.co/datasets/colombia-covid19-coronavirus-departamento/explore?location=4.621900%2C-74.297150%2C5.62 (Open Source information provided by the National Institute of Health of Colombia, https://www.ins.gov.co. Publication date: April 1, 2020). (TIFF) [file pntd.0010228.s005.tiff]

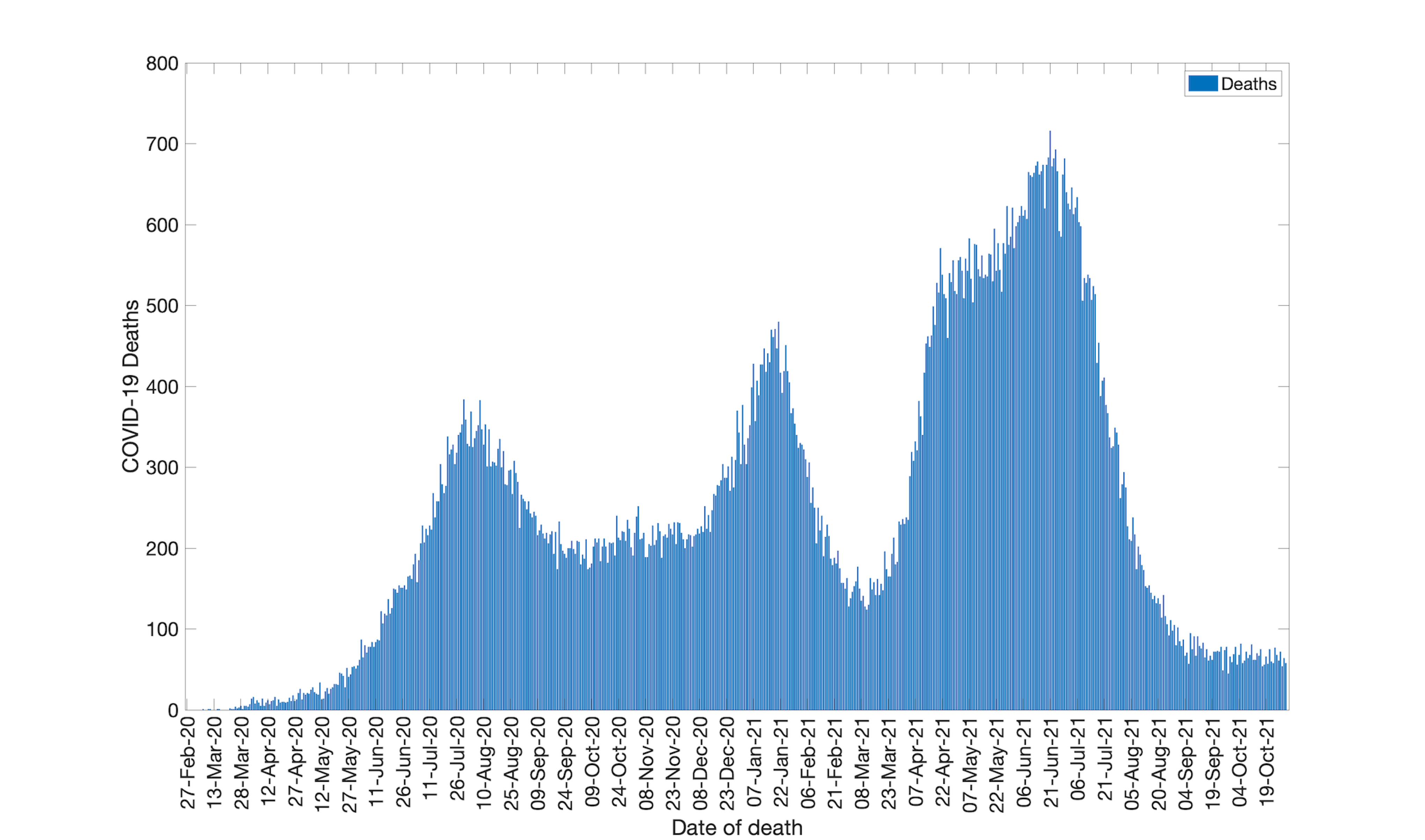

Supplement: S2 Fig — (TIF) [file pntd.0010228.s006.tif]

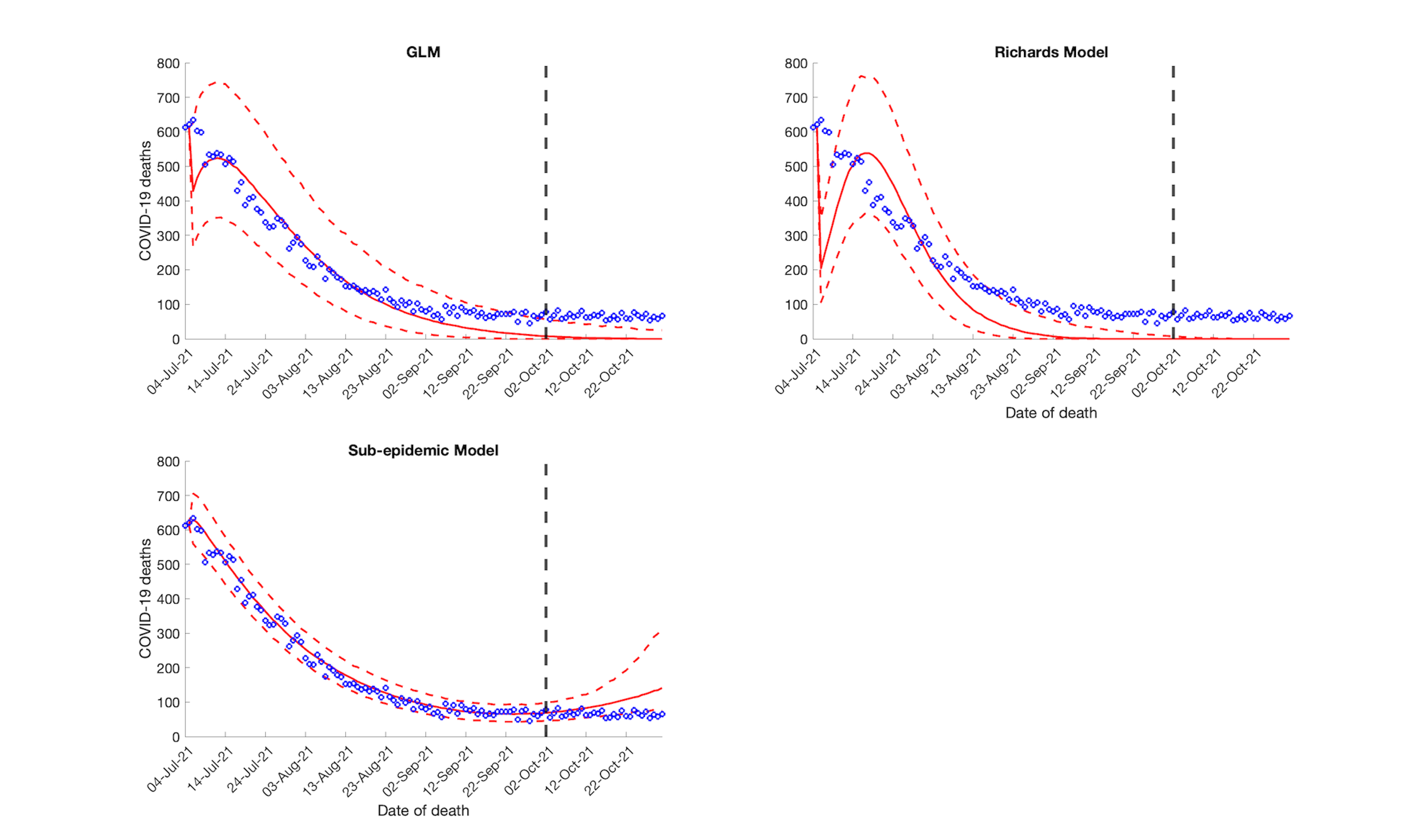

Supplement: S3 Fig — Blue circles correspond to the data points; the solid red line indicates the best model fit, and the red dashed lines represent the 95% prediction interval. The vertical black dashed line represents the time of the start of the forecast period. (TIF) [file pntd.0010228.s007.tif]

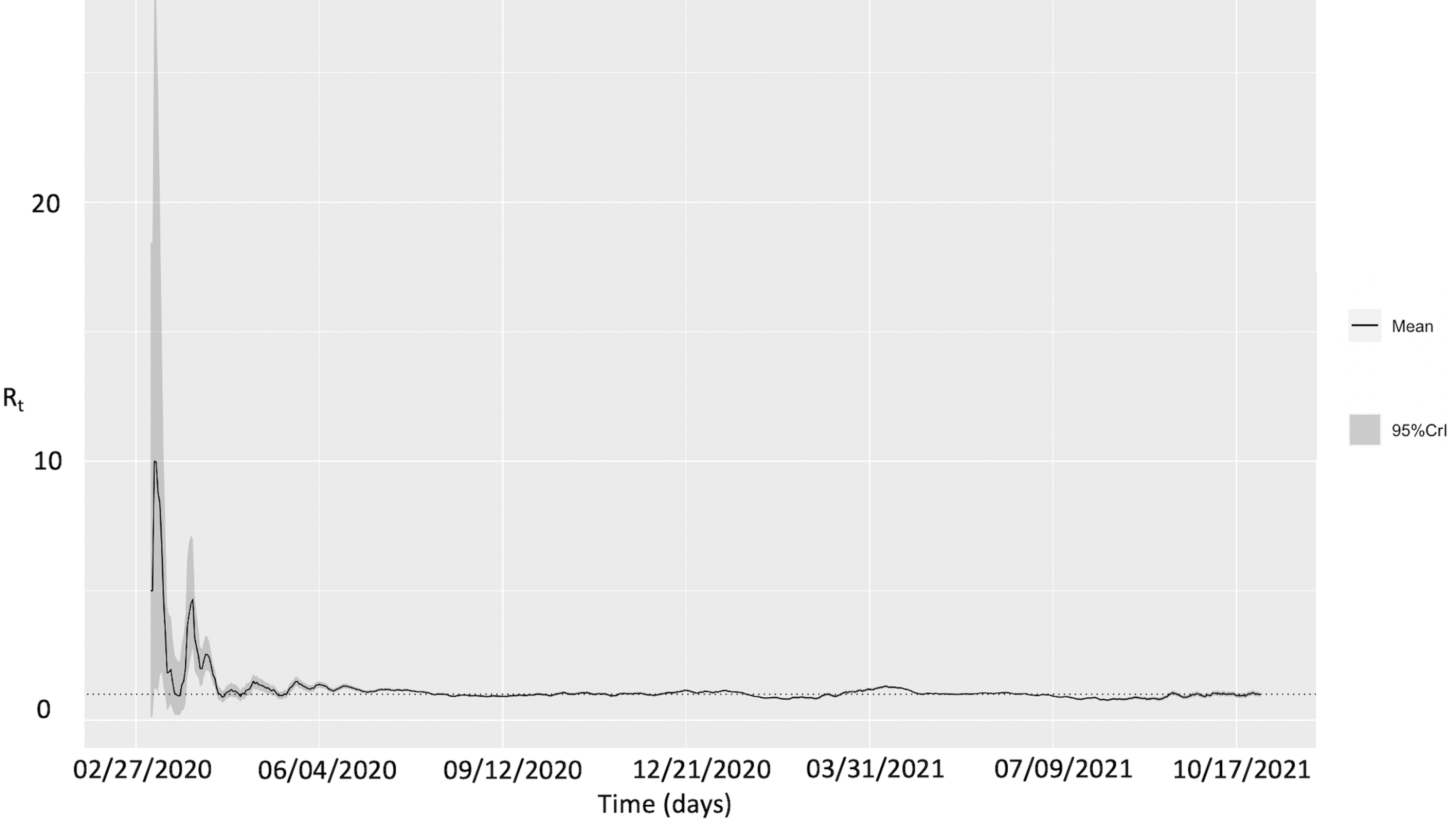

Supplement: S4 Fig — The black solid line represents the mean reproduction number for Colombia and the black shaded area represents the 95% credible interval around it. (TIF) [file pntd.0010228.s008.tif]
